# Antibody-Mediated Protein Knockdown Reveals Distal-less Functions for Eyespots and Parafocal Elements in Butterfly Wing Color Pattern Development

**DOI:** 10.3390/cells13171476

**Published:** 2024-09-02

**Authors:** Yugo Nakazato, Joji M. Otaki

**Affiliations:** The BCPH Unit of Molecular Physiology, Department of Chemistry, Biology and Marine Science, Faculty of Science, University of the Ryukyus, Nishihara, Okinawa 903-0213, Japan

**Keywords:** butterfly wing, color pattern formation, Distal-less, eyespot, nymphalid butterfly, parafocal element, protein delivery, protein knockdown

## Abstract

One of the important genes for eyespot development in butterfly wings is *Distal-less*. Its function has been evaluated via several methods, including CRISPR/Cas9 genome editing. However, functional inhibition may be performed at the right time at the right place using a different method. Here, we used a novel protein delivery method for pupal wing tissues in vivo to inactivate a target protein, Distal-less, with a polyclonal anti-Distal-less antibody using the blue pansy butterfly *Junonia orithya*. We first demonstrated that various antibodies including the anti-Distal-less antibody were delivered to wing epithelial cells in vivo in this species. Treatment with the anti-Distal-less antibody reduced eyespot size, confirming the positive role of Distal-less in eyespot development. The treatment eliminated or deformed a parafocal element, suggesting a positive role of Distal-less in the development of the parafocal element. This result also suggested the integrity of an eyespot and its corresponding parafocal element as the border symmetry system. Taken together, these findings demonstrate that the antibody-mediated protein knockdown method is a useful tool for functional assays of proteins, such as Distal-less, expressed in pupal wing tissues, and that Distal-less functions for eyespots and parafocal elements in butterfly wing color pattern development.

## 1. Introduction

Highly diverse butterfly wing color patterns have attracted intense research interest in evolutionary developmental biology. Although highly diverse, butterfly color patterns have a general pattern called the nymphalid groundplan [1,2,3,4,5,6]. The nymphalid groundplan can be considered a placement of various color pattern elements on a white background. One of the color pattern elements that is probably most conspicuous, not only for birds but also for humans, is the eyespot. An eyespot is a complex (not a simple) element with multiple subelements, including an outer black ring, a core disk, a structural (often white) focus, and a colored ring. The focal area of an eyespot functions as a developmental eyespot organizer, as demonstrated by cautery and transplantation experiments [1,7,8]. Importantly, an eyespot and its accompanying parafocal element (PFE) belong to the same symmetry system called the border symmetry system, according to color pattern analysis of various butterflies [4]. This finding suggests that the parafocal element is the farthest ring of its corresponding eyespot, as specified by the same eyespot focal organizer. If there is a parafocal element but no eyespot in a given compartment, it is likely that the organizer at the focus in that compartment released signals for a parafocal element but then ceased its activity for an eyespot [9]. Nonetheless, PFE is an independent element, probably having its own organizer specified via a serial induction process [6].

Another important feature of the butterfly wing system is that butterfly wing color patterns are composed of scales of various colors. Each scale is produced by a single scale-building cell (scale cell), and each scale is considered a color unit because it may have a single color [1]. Thus, the scale color choices of scale-building cells in an eyespot represent the determination of terminal differentiation to one of the limited fates for scale-building cells in an eyespot in accordance with morphogenic signals from the eyespot organizer. Furthermore, scale cells are largely fixed in the tissue in a nearly two-dimensional plane, which provides researchers with an ideal system to study the mechanisms of fate determination, terminal differentiation, and morphogenesis in response to morphogenic signals from organizers.

Among several genes expressed in butterfly eyespots, the *Distal-less* (*Dll*) gene is one of the most important genes discovered in the earliest molecular studies on butterfly color pattern development [10,11]. *Dll* was first discovered in *Drosophila melanogaster* as a homeodomain transcription factor required for limb development [12,13,14]. *Dll* is also required for wing development in *D. melanogaster* [13,14] and in butterflies [15,16]. In butterfly wings, *Dll* expression signifies the location of future eyespots in the larval wing imaginal disc [10,11]. Eyespot size and *Dll* expression levels are likely linked [17] but are not correlated between seasonal morphs in *Bicyclus anynana* [18]. In *Junonia orithya*, a sexually dimorphic species, females have larger eyespots than males do, but *Dll* expression levels seem to be higher in males than in females [19], suggesting that eyespot size reflects not only *Dll* expression levels but also other factors. Alternatively, *Dll* may have pleiotropic functions in color pattern development. Indeed, *Dll* is expressed in the eyespot focus, eyespot core disk, midlines, wing veins, and background areas during development [11,20,21]. *Dll* and *Notch* together are expressed at the intervein midline [22]. Moreover, *Dll* is upregulated in response to damage [23], which may partly explain damage-induced ectopic spots, although they do not form focal structural scales [24,25,26,27]. On the other hand, *Dll* is unlikely to play a role in black spot formation in pierid butterflies [23,28,29].

In addition to these descriptive studies on expression patterns, functional studies have been performed. *Dll* transgenic and RNAi experiments revealed that *Dll* is positively responsible for eyespot size and black spot formation [30]. Similarly, *Dll* knockout via CRISPR/Cas9 genome editing resulted in a loss-of-eyespot phenotype [16,31]. Furthermore, baculovirus-mediated gene transfer revealed that *Dll* is responsible for elemental induction, although *Dll* alone cannot induce a complete eyespot [32]. On the other hand, a CRISPR/Cas9 genome editing study showed that *Dll* seems to be a negative regulator of eyespot size [33]. Another study, however, noted that CRISPR/Cas9 deletions of different *Dll* exons produced both gains and losses of morphological structures in flies [34]. Similarly, in *B. anynana*, knocking out different *Dll* exons resulted in different eyespot phenotypes, suggesting that *Dll* is a positive regulator of eyespot size [31]. In that study, a high expression of *Dll* resulted in double eyespots in a single compartment [31]. This unique feature of crispants has been explained by computer simulations based on a reaction–diffusion system [31].

Although CRISPR/Cas9 genome editing is a powerful tool for *Dll* functional studies, no single method may perfectly reveal the entire molecular function of *Dll* in vivo. Genome-edited cells may suffer from “side effects” during development even before the stage of color pattern determination. This is because many insect genes, including *Dll,* are used repeatedly in different organs at different time points. Ideally, a gene of interest should be knocked out only at the right time at the right place. Moreover, unexpected splicing and expression may occur in CRISPR/Cas9 genome editing experiments, as noted in previous *Dll* studies [31,34].

To complement previous studies, we here used the protein delivery (transfer) method. We first showed that the protein delivery method that was previously established in the pale grass blue butterfly *Zizeeria maha* [35] was also applicable to the species of interest here, the blue pansy butterfly *J. orithya*. We then delivered the anti-Dll antibody directly to the pupal wing tissue and analyzed color pattern modifications. We obtained interesting color pattern phenotypes that further advanced our understanding of Dll functions in vivo and of the nymphalid groundplan in terms of the status of the parafocal element in the border symmetry system.

## 2. Materials and Methods

### 2.1. Butterflies

Mother butterflies of the blue pansy, *J. orithya*, were caught for egg collection on the Nishihara Campus of the University of the Ryukyus, Okinawa, Japan. The butterflies were confined in a glass tank (300 × 300 × 300 mm) together with a natural host plant, *Phyla nodiflora*, at room temperature (approximately 27 °C) under the L18:D6 cycle. Once hatched, the larvae were reared on another natural host plant, *Plantago asiatica*. After pupation, each pupa with or without the experimental treatment (described below) was confined to an individual container for eclosion. Adult individuals were frozen readily after eclosion.

**Figure 1:**
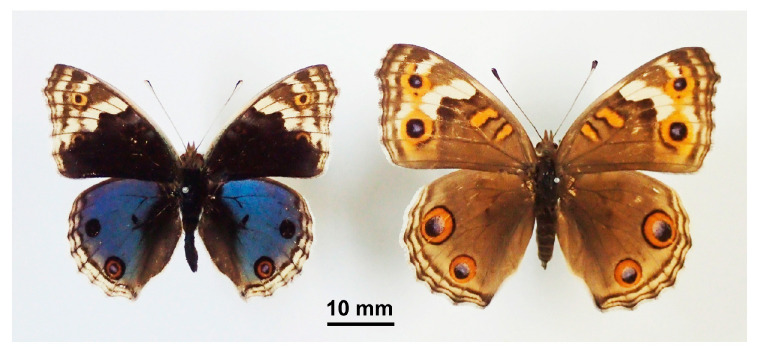
Wing color patterns of *J. orithya*, a sexually dimorphic species. The dorsal hindwings of males (**left**) and females (**right**) have blue and brown background colors, respectively. The present study focused on the anterior eyespot and its surroundings on the dorsal hindwing. These butterfly samples were obtained from Ishigaki-jima Island, Okinawa, Japan.

This butterfly is sexually dimorphic (Figure 1). Males and females have blue and brown background areas, respectively. We focused on the dorsal side of the hindwings. The dorsal hindwing has anterior and posterior eyespots. In males, the anterior eyespot tends to be compromised as a black spot, but we considered it an eyespot in this study.

### 2.2. Antibodies

To test antibody delivery to the pupal wing tissues of the blue pansy butterfly, we used three commercially available monoclonal antibodies: an anti-*Drosophila* axons antibody conjugated with Alexa Fluor 488 (product code: sc-53018 AF488, Santa Cruz Biotechnology, Dallas, TX, USA) (final concentration used: 22.2 μg/mL), an anti-tubulin antibody conjugated with DyLight 550 (product code: NB600-506R, Novus Biologicals, Centennial, CO, USA) (final concentration used: 66.7 μg/mL), and an anti-histone H3 antibody conjugated with DyLight 550 (product code: NBP1-30141R, Novus Biologicals) (final concentration used: 80.0 μg/mL). The anti-tubulin antibody and the anti-*Drosophila* axons antibody were used in a previous study [32].

The polyclonal anti-Dll antibody and anti-spike antibody were raised in the laboratory of Cosmo Bio (Tokyo, Japan) via the Fast Antibody Plus service as described in a previous study [36]. The spike protein is a component of the SARS-CoV-2 virion that is responsible for infection. This anti-spike antibody has been used in a previous study and is called the anti-P1 (peptide 1) antibody [36]. We obtained an anti-spike antibody at a concentration of 280 μg/mL from Cosmo Bio.

The amino acid sequence of the synthetic peptide for immunization for the anti-Dll antibody was determined in reference to the Dll sequence from *J. orithya* (GenBank Accession Number: KR259655, Junonia orithya clone JO-DLL3 Distal-less mRNA, partial cds) as follows from the amino-terminus to the carboxy-terminus: GLSDDPGLRVNGKGKKMRC (19mer), in which the carboxy-terminal cysteine was added for KLH (keyhole limpet hemocyanin) conjugation. Briefly, a rabbit was immunized with this synthetic peptide four times on days 0, 14, 28, and 42. We obtained the anti-Dll antibody at a concentration of 220 μg/mL from Cosmo Bio.

**Figure A1:**
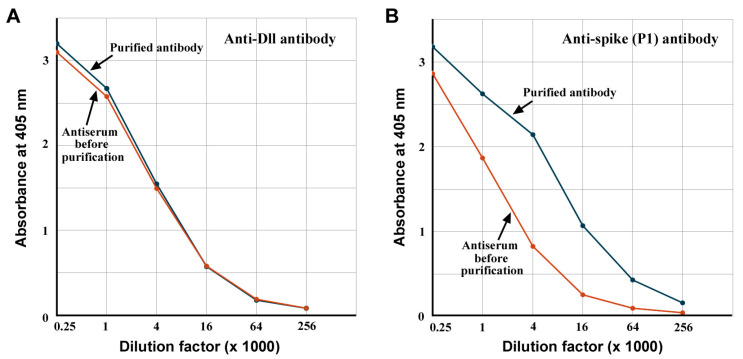
ELISA results of serial dilutions of antibodies before and after purification. (**A**) Anti-Dll antibody. (**B**) Anti-spike antibody.

Both the anti-Dll and anti-spike antibodies were affinity purified via peptide column chromatography. The antibody titers were determined with ELISA (enzyme-linked immunosorbent assay) using anti-rabbit IgG as a secondary antibody conjugated with alkaline phosphatase (Appendix A: Figure A1). For a substrate of alkaline phosphatase, pNPP (*p*-nitrophenyl phosphate) was used, and the absorbance at 405 nm was measured.

**Figure 2:**
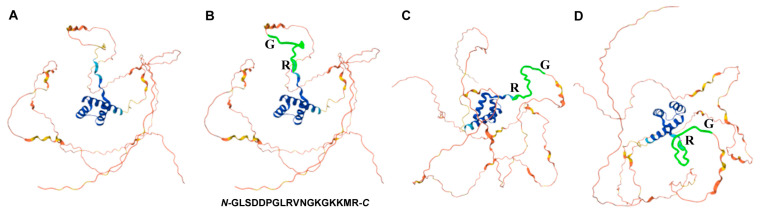
Molecular structure of the *J. coenia* Dll protein as predicted by AlphaFold. (**A**) Predicted Dll protein structure. The colors indicate model confidence levels: cyan: very high, blue: confident, yellow: low, and red: very low. The homeodomain structure (the core helical structure) was predicted with high confidence. (**B**) The same image as (**A**) but with the green highlight for the 18-aa sequence of the synthetic peptide used for immunization. Its sequence is shown at the bottom. The first G (glycine) and the last R (arginine) of the peptide sequence are indicated adjacent to the structure. (**C**,**D**) Predicted *J. coenia* Dll protein structure from different angles. Locations of the first G and the last R of the peptide sequence are indicated.

The UniProt Knowledgebase (UniProtKB) (https://www.uniprot.org/uniprotkb) (accessed on 19 June 2024) and its associated AlphaFold structure prediction of Dll proteins from three species, *D. melanogaster* (P20009·DLL_DROME), *J. coenia* (Q95VX3·Q95VX3_JUNCO), and *J. orithya* (A0A172JH25·A0A172JH25_JUNOR) (accessed on 19 June 2024), indicated that the synthetic peptide epitope was located at the beginning of the homeobox of the Dll protein and was mostly not structured (Figure 2). This region is relatively hydrophilic and appears to be accessible from the outside, which is likely suitable for an antibody target.

To observe the delivery of the anti-Dll antibody to the pupal wing tissues, the anti-Dll antibody was condensed (3×) and conjugated with fluorescein using the Fluorescein Labeling Kit -NH_2_ (Dojindo Laboratories, Kumamoto, Japan). The conjugated antibody was delivered via the sandwich and injection methods, and its fluorescent signals were observed as described below.

### 2.3. Protein Delivery for Microscopic Observations

According to a previous study [35], we used ProteoCarry (Funakoshi, Tokyo, Japan), which is a proprietary peptide reagent [37] (accessed on 14 June 2024), for protein delivery to cells. We used a volumetric ratio of 8:1 for ProteoCarry:antibody throughout this study. For delivery, we used both the sandwich method and the injection method [35]. For the former, the forewing was physically lifted using forceps 20–50 min postpupation for commercial antibodies and 15–40 min postpupation for anti-Dll and anti-spike antibodies. The distal half of the forewing was lifted. A droplet of the mixture of ProteoCarry and an antibody was placed on the surface of the dorsal hindwing near the anterior eyespot and its vicinity, and the lifted forewing was carefully placed back to the original position. For confocal microscopy, the volume of the sandwiched droplet was 20 μL, and the loading duration was 60 min. Afterwards, the dorsal hindwing was washed with 200 μL of Insect Ringer solution. After removal of the Insect Ringer solution, the fluorescent indicators Hoechst 33342 (Dojindo Laboratories), BODIPY FL C_5_-ceramide complexed to BSA (Thermo Fisher Scientific, Tokyo, Japan), and/or MitoRed (Dojindo Laboratories) were then loaded for 60 min. Again, the dorsal hindwing was washed with 200 μL of Insect Ringer solution. The hindwing was placed on a glass plate, and the entire pupa was covered with plastic film to prevent the pupa from drying.

For the injection method, the forewing was lifted, and Hoechst 33342, BODIPY FL C_5_-ceramide, and/or MitoRed were loaded, as in the sandwich method. The ProteoCarry-antibody mixture (2.0 μL) was then injected into the abdomen of each pupa. The dorsal hindwing was then observed via the same method as the sandwich method.

We used a Nikon A1+ ECLIPSE Ti confocal microscope system (Tokyo, Japan) for observations at the cellular and subcellular levels. The hardware was driven by NIS-Elements AR 4.40.00 (Nikon). Optical cross sections were made every 0.4 μm or 0.2 μm.

### 2.4. Liquid Distribution

The distribution of liquid droplets of an antibody solution when applied in the sandwich method was examined using an anti-tubulin antibody conjugated with DyLight 550. The forewing was lifted 15–40 min postpupation as described above. After that, 0.6 μL of the anti-tubulin antibody was placed on the surface of the hindwing. A small piece of plastic film was placed over the exposed surface of the hindwing. The distribution of the antibody-containing liquid on the surface of the hindwing was observed using a Nikon multipurpose zoom microscope AZ100 with a Nikon digital sight DS-5Mc CCD camera.

### 2.5. Protein Delivery for Color Pattern Modifications

For the delivery of anti-Dll and anti-spike antibodies for color pattern modifications in the sandwich method, the pupal forewing was lifted 15–40 min after pupation. The volume of the ProteoCarry-antibody mixture placed on the surface of the hindwing was 0.6 μL. The ProteoCarry:antibody ratio was 8:1, as described above. The forewing lift operation was performed as described above. For the injection method, the ProteoCarry-antibody mixture (2.0 μL) was injected into the abdomen of each pupa. After the treatment, each pupa was individually placed in a container until eclosion. After eclosion, the adult butterflies were frozen immediately for inspection of wing color pattern changes.

### 2.6. Immunohistochemistry

The current immunohistochemistry protocol was similar to but modified from that of the previous study [32]. Pupae were lightly anesthetized on ice immediately after pupation (within 1 h postpupation). Under a dissection microscope, a forewing was first lifted with forceps. Then, an underlying hindwing was similarly lifted and cut out with scissors at the base. The hindwing tissue was placed and expanded on a glass slide to make the doral side up. Hemolymph from the isolated wing tissue was cleaned with Kimwipe. The tissue was quickly dried with a Sharp Plasmacluster room dehumidifier CV-J71 (Osaka, Japan) for approximately 10 min.

The partially dried tissues were fixed with 4% paraformaldehyde and 1.5% glutaraldehyde in 0.1 M sodium cacodylate solution (pH 7.4) at 4 °C for two days. After the fixation, the tissues were incubated with 1% hydrogen peroxide in PBS for 1.5 h to quench endogenous peroxidase activity. A blocking process was performed with 0.5% NP-40 (BioVision, Abcam, Cambridge, UK) and 1.5% normal goat serum (Vector Laboratories, Burlingame, CA, USA) in PBS for 4.5 h. The tissues were then incubated with the anti-Dll antibody (1:100 dilution) overnight. The tissues were then incubated with biotinylated anti-rabbit IgG made in goat (1:200 dilution) (Vector Laboratories) for 4 h. The tissues were then treated with VectaStain Elite ABC Kit (Vector Laboratories) and subsequently with DAB Peroxidase Substrate Kit (Vector Laboratories). Finally, the tissues were mounted with Ultramount Aqueous Permanent Mounting Medium (DAKO, Agilent, Santa Clara, CA, USA). For observations and image acquisition, we used a Nikon multipurpose zoom microscope AZ100 with a Nikon digital sight DS-5Mc CCD camera.

### 2.7. Wing Images and Eyespot Area Measurements

Digital wing images were obtained using a Keyence Digital Microscope VHX-7000 (Osaka, Japan). The area values of the anterior eyespots were obtained using the automatic area measurement function of the same microscope in brightness mode. The covered areas were visually confirmed.

### 2.8. Statistical Analyses

To compare the eyespot size between the treated (left) and untreated (right) wings in the sandwich method, we performed a two-sided paired *t* test using Microsoft Excel (Microsoft Office 365). In addition, the left eyespot size was divided by the right eyespot size of the same individual. This left/right ratio was compared between the anti-Dll antibody-treated and anti-spike antibody-treated butterflies. For this analysis, we performed a two-sided unpaired *t* test using Microsoft Excel after excluding individuals in which the left/right ratio was zero. To compare the numbers of modified individuals between the anti-Dll and anti-spike treatments, a *χ*^2^ test (in the sandwich method) or a Fisher’s exact test (in the injection method) was performed using JSTAT Version 16.1 (Yokohama, Japan).

## 3. Results

### 3.1. Antibodies Were Delivered to Cells via the Sandwich Method

**Figure 3:**
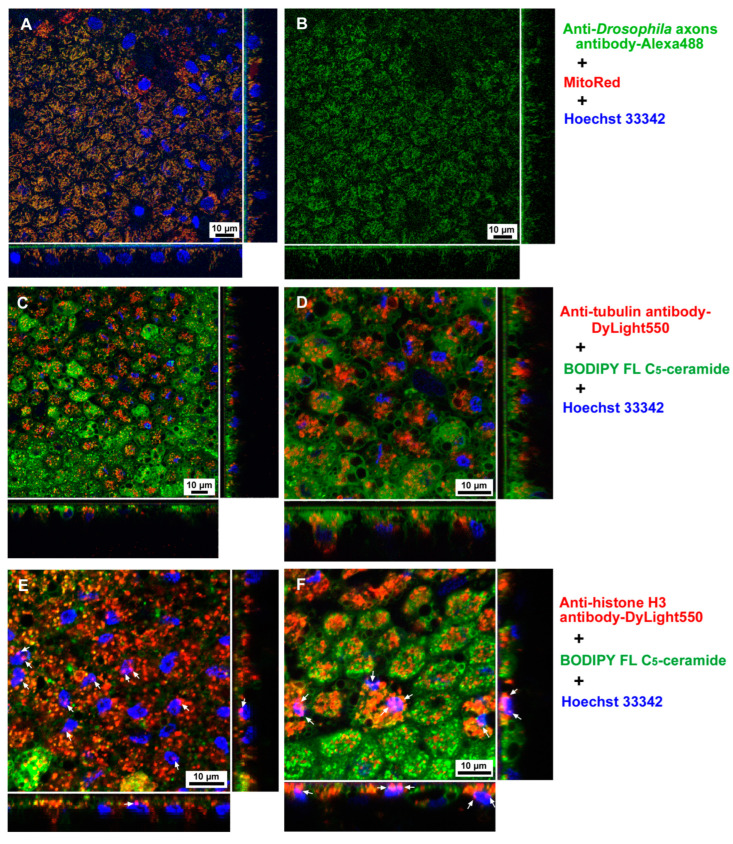
Antibody delivery to butterfly pupal wing tissue (dorsal hindwing) via the sandwich method. Fluorescent colors are indicated by colors of letters on the right. (**A**) Anti-*Drosophila* axons antibody conjugated with Alexa 488. (**B**) The same visual field as in (**A**) but with only fluorescent signals from the anti-*Drosophila* axons antibody. Most green signals are colocalized with red signals. (**C**,**D**) Anti-tubulin antibody conjugated with DyLight 550. (**E**,**F**) Anti-histone H3 antibody conjugated with DyLight 550. These two images are from different individuals. There are purple signals from nuclei (arrows), indicating the localization of the anti-histone H3 antibody in the nucleus.

We first tested whether commercial antibodies can be delivered to epithelial (epidermal) cells of pupal wing tissues in vivo in *J. orithya* via the sandwich method. To this end, we tested three antibodies for protein delivery: an anti-*Drsosophila* axons antibody, an anti-tubulin antibody, and an anti-histone H3 antibody. In all three treatments, not all individuals showed successful protein delivery, and in the individuals with successful delivery, the distributions of fluorescent cells were patchy in the wing tissue, as in a previous study [35]. In the case of the anti-*Drosophila* axons antibody, the delivery rate on the individual basis was 58% (positives *n* = 7 among the treated *n* = 12). At the cellular level, anti-*Drosophila* axons antibody fluorescent signals were almost perfectly colocalized with mitochondrial MitoRed signals in the cells (Figure 3A,B). In the case of the anti-tubulin antibody, the delivery rate was 50% (positives *n* = 5 among the treated *n* = 10). Fluorescent signals from the anti-tubulin antibody were also detected in the cells (Figure 3C,D). In this case, many cells were full of red signals, but many fluorescent signals also seemed to be vesicular and may have been located in lysosomes. In the case of the anti-histone H3 antibody, the delivery rate was 75% (positives *n* = 9 among the treated *n* = 12). Fluorescent signals from the anti-histone H3 antibody were abundantly detected in the cells, as in the case of the anti-tubulin antibody. Importantly, the red signals from the anti-histone H3 antibody clearly colocalized with the blue signals from Hoechst 33342, an indicator of nuclei, showing purple signals (colocalized *n* = 8) (Figure 3E,F). The anti-histone H3 antibody signals seemed to be restricted in an apical portion of the nucleus (Figure 3E,F). In contrast, no such nuclear colocalization was observed with the anti-*Drosophila* axons antibody (Figure 3A,B) or the anti-tubulin antibody (Figure 3C,D). These results demonstrated that the anti-histone H3 antibody bound to histone H3 in the nucleus.

**Figure 4:**
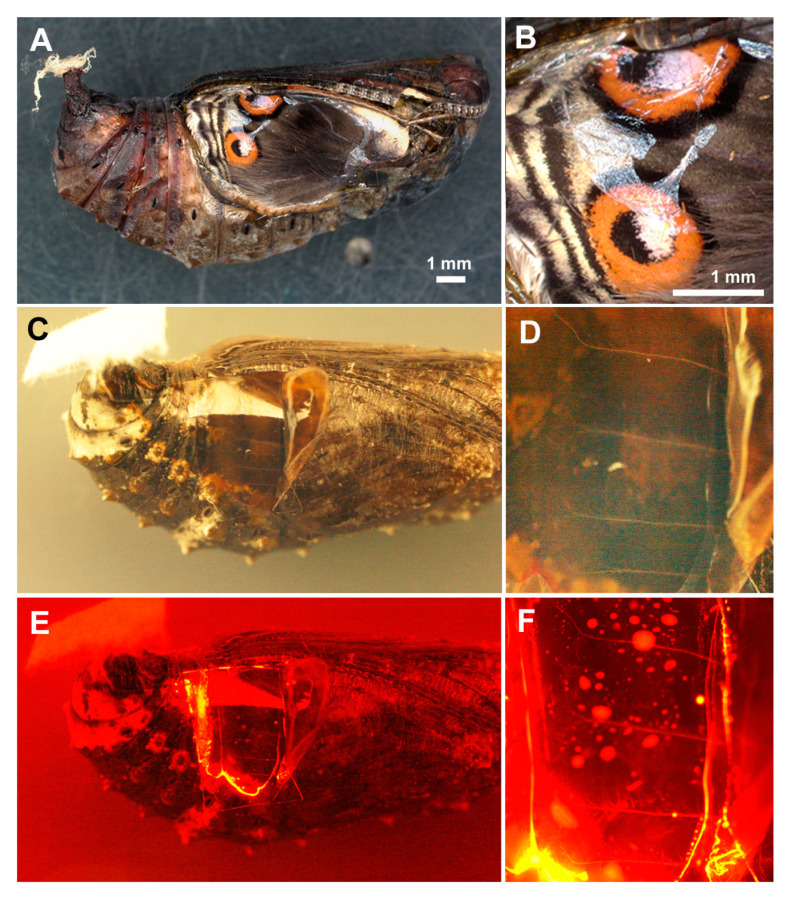
Liquid distribution on the surface of the dorsal hindwing after the anti-tubulin antibody conjugated with DyLight 550 was sandwiched. (**A**) A pupa before eclosion. The forewing was removed, and the dorsal hindwing was exposed. Anterior and posterior eyespots are clearly visible. (**B**) Magnification of the eyespots in (**A**). (**C**) A pupa with the forewing lifted and the hindwing covered with a piece of plastic film under visible white light. The anti-tubulin antibody is sandwiched between the hindwing and plastic film. (**D**) Magnification of (**C**). The dorsal hindwing has wing veins (tracheae). (**E**) The same visual field as (**C**) under green excitation light. (**F**) Magnification of (**E**). There are numerous red-fluorescing liquid droplets.

Because the delivery rate was not close to 100% and because the positive cells were not evenly distributed in the delivered cases, we examined the behavior of the antibody-containing liquid after being sandwiched. The position of the application was on the anterior eyespot and its adjacent background area (Figure 4A,B). When a piece of plastic wrap was used to sandwich the mixture (Figure 4C,D), we observed that the liquid formed numerous droplets of various sizes and did not show an even distribution, and much of the antibody was found along the edge of the wing (*n* = 4) (Figure 4E,F), suggesting that this behavior of the liquid may be a reason for the imperfect delivery rates and uneven antibody delivery reported in the previous study [35] and the present study.

### 3.2. Antibodies Were Delivered to Cells via the Injection Method

**Figure 5:**
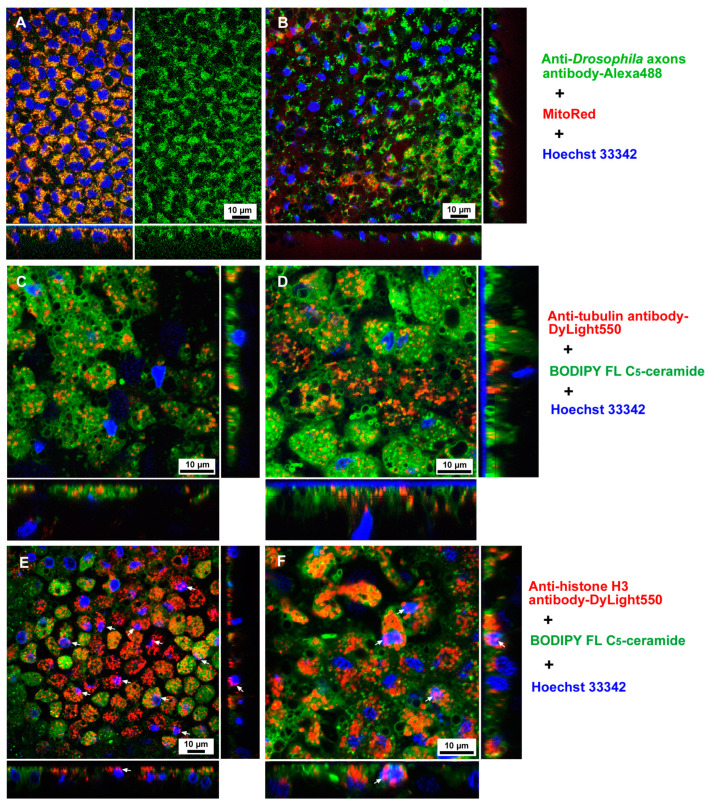
Antibody delivery to butterfly pupal wing tissue (dorsal hindwing) via the injection method. Fluorescent colors are indicated by colors of letters on the right. A pair of panels was obtained from different individuals. (**A**,**B**) Anti-*Drosophila* axons antibody conjugated with Alexa 488. Most green signals are colocalized with red signals in (**A**), producing orangish or yellowish signals when three channels of fluorescent colors are observed simultaneously, as shown in the left panel of (**A**). A single channel image of the anti-*Drosophila* axons antibody conjugated with Alexa 488 is shown in the right panel of (**A**). (**C**,**D**) Anti-tubulin antibody conjugated with DyLight 550. (**E**,**F**) Anti-histone H3 antibody conjugated with DyLight 550. There are many purple signals from the nuclei (arrows), indicating the localization of the anti-histone H3 antibody in the nucleus.

We then performed the injection method to deliver the three commercial antibodies used above into epithelial cells. We obtained results similar to those of the sandwich method. In the case of the anti-*Drosophila* axons antibody, the delivery rate was 100% (positives *n* = 11 among the treated *n* = 11), and we observed almost completely overlapping signals of the antibody with MitoRed in most individuals (*n* = 9) (Figure 5A), but the overlap was incomplete in two individuals (Figure 5B). In the case of the anti-tubulin antibody, the delivery rate was 60% (positives *n* = 6 among the treated *n* = 10), and we observed strong signals of the antibody from epithelial cells (Figure 5C,D). In the case of the anti-histone H3 antibody, the delivery rate was 40% (positives *n* = 4 among the treated *n* = 10), and we observed extensive signals of the antibody from epithelial cells (Figure 5E,F). Notably, some of the red signals from this antibody were also located in the nucleus, producing purple signals due to colocalization with the blue signals from Hoechst 33342 (colocalized *n* = 4). Purple signals were localized not only at the apical portion but also at the basal portion of the nucleus (Figure 5E,F).

### 3.3. Anti-Dll Antibody Was Delivered to Cells

**Figure 6:**
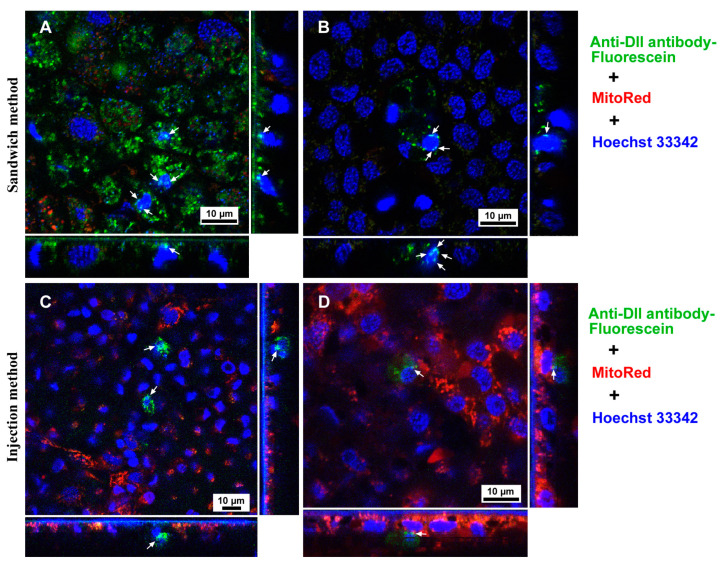
Delivery of the anti-Dll antibody to butterfly pupal wing tissue (dorsal hindwing) via the sandwich and injection methods. Fluorescent colors are indicated by colors of letters on the right. There are many blue–green signals from the nuclei (arrows), indicating the localization of the anti-Dll antibody in the nucleus. A pair of panels was obtained from different individuals. (**A**,**B**) Sandwich method. (**C**,**D**) Injection method.

To demonstrate that the anti-Dll antibody can be delivered to wing epithelial cells in vivo, we labeled the anti-Dll antibody with fluorescein and performed both the sandwich and injection methods. In the sandwich method, the delivery rate was 100% on the individual basis (positives *n* = 9 among the treated *n* = 9) (Figure 6A,B). Among them, we detected positive signals of the anti-Dll antibody colocalized with the Hoechst nuclear signals (colocalized *n* = 6). In the injection method, the delivery rate was 88% (positives *n* = 7 among the treated *n* = 8) (Figure 6C,D). Among them, we detected positive signals of the anti-Dll antibody colocalized with the Hoechst nuclear signals (colocalized *n* = 3). In both methods, the detected nuclear localization signals were relatively weak, compared with those of the anti-histone H3 antibody, which may be simply because the number of Dll protein molecules is limited in a cell, considering that Dll is a transcription factor.

### 3.4. Dll Expression at the Prospective Eyespot Focus in the Pupal Wing Tissue

**Figure 7:**
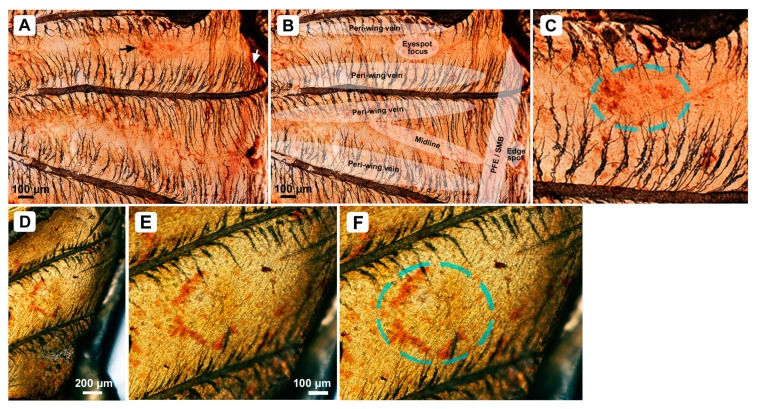
Immunohistochemical analysis of Dll in the pupal wing tissue (dorsal hindwing) immediately after pupation. (**A**) Two wing compartments with and without a prospective eyespot focus. The prospective anterior eyespot focal area shows positive staining (black arrow). The prospective PFE/SMB also shows positive staining (white arrow). (**B**) Annotations of the positive areas shown in (**A**). (**C**) Enlargement of the prospective anterior eyespot focal area shown in (**A**), indicated by a broken circle. (**D**) Prospective anterior eyespot focal area of an individual different from (**A**–**C**), showing a ring-like pattern. (**E**,**F**) Enlargement of (**D**). The prospective anterior eyespot focal area is indicated by a broken circle in (**F**).

We immunohistochemically confirmed that Dll was expressed at the prospective eyespot focal area in the pupal wing tissue using the anti-Dll antibody (*n* = 4) (Figure 7A–D). The Dll-positive signals showed a dotted or ring-like pattern. We did not clearly confirm the Dll-positive signals in the eyespot-less imaginary focus, but a part of the midline seemed to be positive (Figure 7A,B). Peripheral staining possibly for PFE or SMB was also detected together with a strong staining of a middle point along the wing edge, which corresponds to the edge spot on the pupal cuticle (Figure 7A,B). The Dll-positive signals along the wing veins were also detected in this study (Figure 7A,B). The potential Dll-positive signals in the background area were difficult to detect clearly because of the background noise.

### 3.5. Anti-Dll Antibody Reduced the Eyespot Size via the Sandwich Method

**Figure 8:**
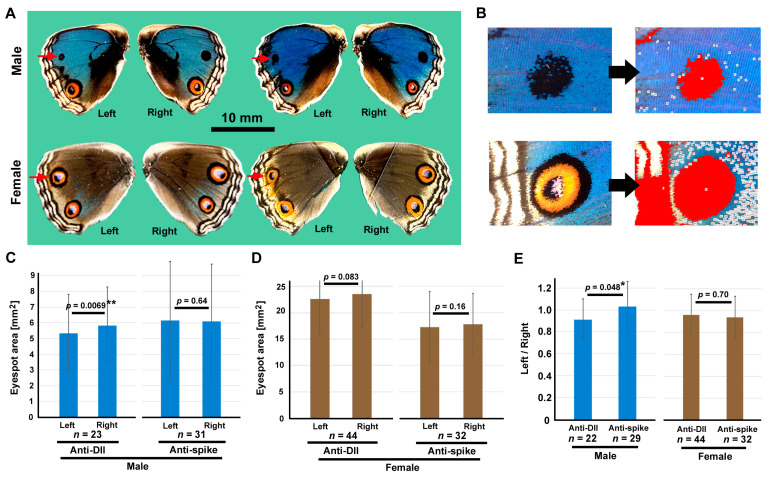
Effect of anti-Dll antibody delivery on eyespot size. (**A**) Representative male (top) and female (bottom) hindwings treated with an anti-Dll antibody. The anterior eyespot (red arrows) in the left hindwing was compared with the untreated anterior eyespot in the right hindwing within a single individual. (**B**) Two examples of an automatic processing for the eyespot area values from digital images. (**C**) Comparison of the eyespot area values in males (two-sided paired *t* test). **: *p* < 0.01. (**D**) Comparison of the eyespot area values in females (two-sided paired *t* test). (**E**) Left/right ratio comparisons between the anti-Dll and anti-spike antibodies in males (left) and females (right) (two-sided unpaired *t* test). *: *p* < 0.05.

We performed the delivery of the anti-Dll antibody via the sandwich method because the antibody delivery to the nucleus was demonstrated in the previous section, and we obtained treated adult individuals (*n* = 67) (Supplementary Files S1 and S2). For comparison, we also performed the delivery of the anti-spike antibody and obtained control individuals (*n* = 63) (Supplementary Files S3 and S4). The anti-spike antibody was not supposed to interact with proteins present in *J. orithya*. The survival rates of both treatments were high and similar to each other, demonstrating the validity of the experimental comparison (Table 1). To examine the effect of the antibodies on eyespot size, we focused on the anterior eyespot, on which the ProteoCarry-antibody mixture was placed (Figure 8A). We measured the area values of the eyespots using the automatic function of the microscope (Figure 8B).

Between the treated (left) and untreated (right) eyespots, the treated eyespots were significantly smaller than the untreated eyespots when the anti-Dll antibody was applied to the males (*p* = 0.0069) (Figure 8C, left). When the anti-spike antibody was applied, no significant difference was found between the treated and untreated eyespots in males (*p* = 0.64) (Figure 8C, right). In females, the size differences in the anti-Dll antibody (*p* = 0.083) and anti-spike antibody (*p* = 0.16) were not statistically significant between the treated and untreated eyespots (Figure 8D, left), although the *p*-value was close to the significance level for the anti-Dll antibody. A comparison of the left/right ratios between the anti-Dll and anti-spike antibodies was also significant in males (*p* = 0.048) but not in females (*p* = 0.70) (Figure 8E).

### 3.6. Anti-Dll Antibody Induced Modifications of Parafocal Elements

**Figure 9:**
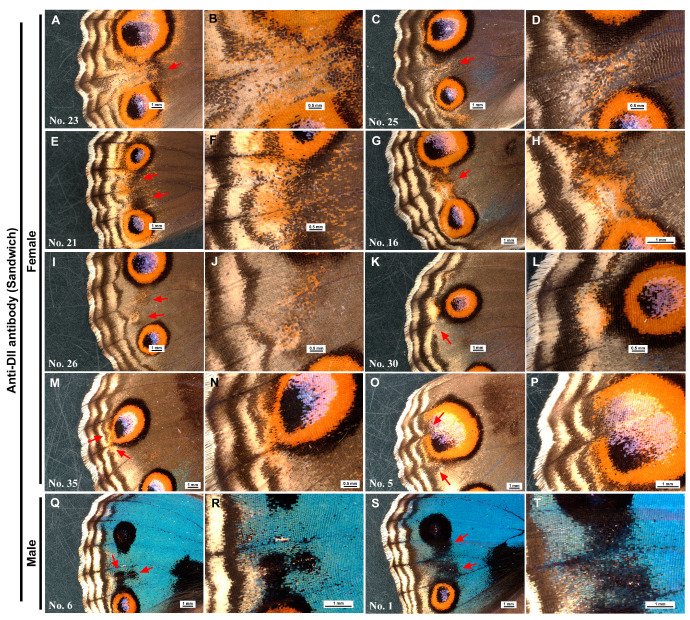
Color pattern modifications induced by the application of the anti-Dll antibody via the sandwich method. In a pair of panels, an area indicated by an arrow or arrows in the left panel is magnified in the right panel. Individual numbers are indicated in the first panel of an individual (Supplementary Files S1 and S2). (**A**,**B**) A female individual with invagination of parafocal element (PFE). The PFE is curved along two adjacent eyespots, and the submarginal band (SMB) is expanded proximally. Additionally, there may be a small ectopic eyespot (arrow). (**C**,**D**) A female individual with a phenotype similar to (**A**,**B**). (**E**,**F**) A female individual with possible knockdown of eyespot-less areas, including the midline and the imaginary focal areas. The PFE is curved, although less intense than in the previous cases, and black spots (arrows) emerge. (**G**,**H**) A female individual with an ectopic black and orange pattern between the two eyespots. The PFE is not deformed. (**I**,**J**) A female individual with small ectopic black and orange patterns (arrows) between the two eyespots. (**K**,**L**) A female individual with PFE deletion (arrow) and a thickened SMB. (**M**,**N**) A female individual showing a partial loss of PFE (arrow) and PFE invagination (arrow) toward the eyespot focus. (**O**,**P**) A female individual showing PFE invaginations (arrows) toward eyespot foci. (**Q**,**R**) A male individual with two ectopic black spots (arrows) in a single compartment. (**S**,**T**) A male individual with ectopic black areas (arrows) in two compartments.

In addition to the size reduction shown above, the anti-Dll-antibody treatment resulted in a few types of color pattern changes (Figure 9; Supplementary Files S1 and S2). In females, an outstanding modification occurred, in which a PFE may be pulled to the imaginary focus (Figure 9A,B). A similar invagination phenotype was also observed in three additional individuals (Figure 9C–F). In other individuals, ectopic patterns involving black and orange bands were observed between the two eyespots (Figure 9G–J). Similar modifications were observed in the other five individuals. One individual exhibited a loss-of-PFE phenotype (Figure 9K,L). In this individual, almost half of the PFE in a compartment was not present. The submarginal band (SMB) near the deleted PFE was thickened. Partial loss of PEF was also observed in the other four individuals. In one individual, we observed a partial loss of PFE combined with a possible invagination of PFE toward the eyespot core (Figure 9M,N). Similar modifications occurred in two additional individuals without a loss of PFE (Figure 9O,P). In males, black spots between the two eyespots and potential invagination of the PFE were observed (Figure 9Q–T). In total, 23 individuals (males *n* = 6 and females *n* = 17) presented modifications in color patterns among the 67 individuals with successful eclosion, yielding a 34.3% modification rate (Table 1).

**Figure 10:**
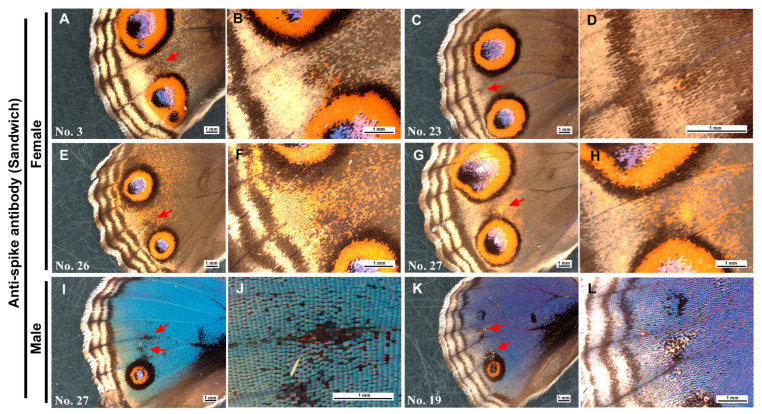
Color pattern modifications induced by the application of the anti-spike antibody via the sandwich method. In a pair of panels, an area indicated by an arrow or arrows in the left panel is magnified in the right panel. Individual numbers are indicated in the first panel of an individual (Supplementary Files S3 and S4). (**A**,**B**) A female individual with an ectopic color pattern (arrow) in the eyespot-less imaginary focal area. (**C**,**D**) A female individual with an ectopic color pattern, similar to (**A**,**B**), but outside the imaginary focal area. (**E**,**F**) A female individual with a possible ectopic color pattern (arrow). (**G**,**H**) A female individual with an ectopic color pattern (arrow) near the imaginary focal area. (**I**,**J**) A male individual with two ectopic black areas (arrows). Some scales appear to be removed. (**K**,**L**) A male individual with two ectopic black areas (arrows). They are likely associated with the removal of scales.

In contrast, only minor color pattern modifications were observed in the wings treated with the anti-spike antibody (Figure 10; Supplementary Files S3 and S4). In some female individuals, minor ectopic patterns were present between the two eyespots (Figure 10A–H). Similar ectopic black areas were also present in some male individuals, but the black areas appeared to indicate the removal of cover scales and/or ground scales (Figure 10I–L). In total, among the 63 individuals with successful eclosion, 8 individuals (males *n* = 2 and females *n* = 5) presented minor changes in color patterns, yielding an 11.1% modification rate (Table 1). Hence, the modification rate of the anti-Dll antibody (34.3%) was 3.1-fold greater than that of the anti-spike antibody, aside from the quality of the modifications. Indeed, the numbers of modified and nonmodified individuals were significantly different between the two antibody treatment groups (*χ*^2^ test; *p* = 0.0003 without adjustment; *p* = 0.0006 with Yates adjustment).

### 3.7. Anti-Dll Antibody Affected Eyespot Size and Scale Development via the Injection Method

**Figure 11:**
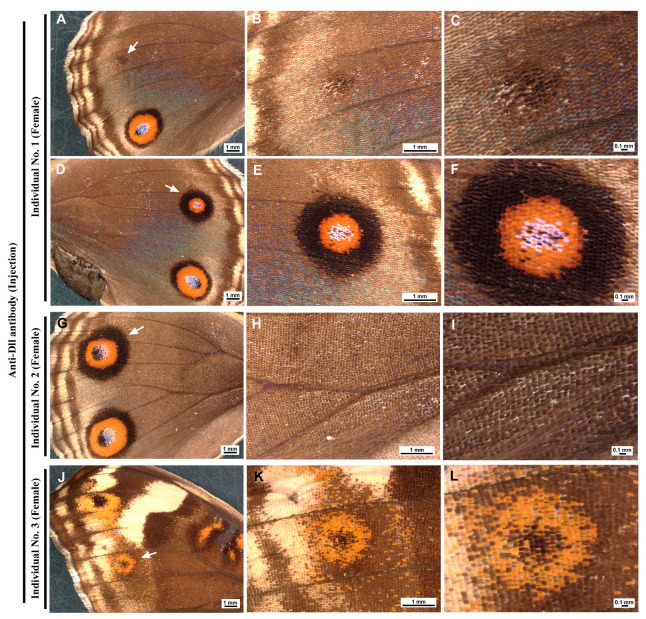
Color pattern modifications induced by the application of the anti-Dll antibody via the injection method. Three panels in the same row indicate a serial magnification of the first panel. Individual numbers are indicated on the left (Supplementary Files S5 and S6). (**A**–**C**) A female individual with a remnant eyespot (arrow) on the left hindwing. Note scarcity of scales. (**D**–**F**) Same female individual with an eyespot size reduction on the right hindwing. (**G**–**I**) A female individual with a potentially affected eyespot (arrow) and impaired scale development in the background. (**J**–**L**) A female individual with a reduced eyespot (arrow) on the forewing.

In addition to the sandwich method described above, we used the injection method for the delivery of the anti-Dll antibody to wing epithelial cells. Among the individuals treated (*n* = 12), most individuals survived (*n* = 10), with a survival rate of 83.3% (Table 2; Supplementary File S5). Two individuals had very small eyespots, suggesting that the anti-Dll antibody treatment caused a reduction in the size of the eyespots (Figure 11A–F,J–L). Notably, two individuals exhibited impaired scale development (Figure 11A–I). Both showed an eyespot with a relatively wide outer black ring. Including these individuals with the impaired scale development, in total, three individuals presented modifications, resulting in a modification rate of 30.0%. In contrast, among the individuals treated with the anti-spike antibody (*n* = 16), most individuals survived (*n* = 15), with a survival rate of 93.7% (Table 2; Supplementary File S6). None of the treated individuals presented any changes in color patterns or scale development. The difference in the number of modified and nonmodified individuals between the two antibody treatment groups was close to significance (Fisher’s exact test; *p* = 0.052).

## 4. Discussion

In the present study, we first attempted to deliver three commercial antibodies to the epithelial cells of the pupal wing tissues of *J. orithya*. The antibody delivery pattern via the sandwich method appeared to vary somewhat from individual to individual, and the delivery rate was not close to 100%, although the ProteoCarry-antibody mixture was precisely placed at the position of the prospective anterior eyespot or in its vicinity. These observations were also reported in a previous study [35] and likely stem from the fact that the mixture did not dissipate evenly on the surface of the wing tissue; the antibody-containing liquid formed many droplets and “gathered” at the wing edges (Figure 4). We could not precisely control the distribution pattern of the droplets, but we imagine that the cells under such droplets were stained well. Roughly, similar delivery rates were obtained via the sandwich and injection methods. Consistently, the delivery rates of the anti-Dll antibody via the sandwich and injection methods were also similar. At the cellular level, we detected fluorescent signals from the three commercial antibodies, and we concluded that antibody delivery was successful in *J. orithya* despite the above concerns. Notably, the nuclei were stained well with the anti-histone H3 antibody via both the sandwich and injection methods, demonstrating that an antibody can be delivered to the nuclei in this system. We then demonstrated that the anti-Dll antibody was indeed delivered to cells and to the nuclei.

Antibody-mediated knockdown of a protein of interest is a new methodology in butterfly biology. This method is relatively easy to perform with no special equipment as long as an antibody is available, but in addition to the staining variability and the imperfect delivery rate, there are some drawbacks that should be improved upon in the future. First, this method requires careful manipulation of the pupae for sandwiching the mixture. This is because physical damage may induce ectopic patterns [24,25,26,27]. The forewing lift operation alone may reduce or enlarge eyespots [38]. For this reason, even the anti-spike antibody treatment (control) had minor effects on 11.1% of the individuals. The injection method was easier than the sandwich method with no such concerns and with similar delivery rates, but it suffered from a difficulty in comparisons between the right and left wings. Second, occasionally, the cells (or scales) that incorporated antibody molecules in the adult wings were not easily identified. This could be judged only by color pattern modifications in reference to the untreated wings.

Given these points for future improvements, we believe that the current method is reliable enough to understand the function of Dll or any other protein molecules expressed in the pupal wing tissue. Fortunately, the color pattern modifications observed in the anti-spike antibody-treated individuals were minor and distinguishable from those observed in the anti-Dll antibody-treated individuals. In the future, it would be important to use the new protein knockdown method, considering its advantages and disadvantages over other methods. In addition to the sandwich and injection methods, other delivery methods may be invented in the future to improve the delivery rates.

We confirmed immunohistochemically that Dll is expressed at the prospective eyespot focal area, along the wing veins, in the midline, and in the peripheral area (PFE or SMB) in the pupal wing tissue, as shown in the larval wing imaginal discs in other studies [10,11,20,21]. We could not clearly detect Dll expression at the eyespot-less imaginary focus or in the background area. Upon delivery of the anti-Dll antibody via both the sandwich and injection methods, we observed a reduction in eyespot size, indicating that the Dll functions to establish eyespot signals at the prospective eyespot foci. We also observed pattern changes in the PFEs via the sandwich method. Additionally, the background area might have been affected by the anti-Dll antibody.

**Figure 12:**
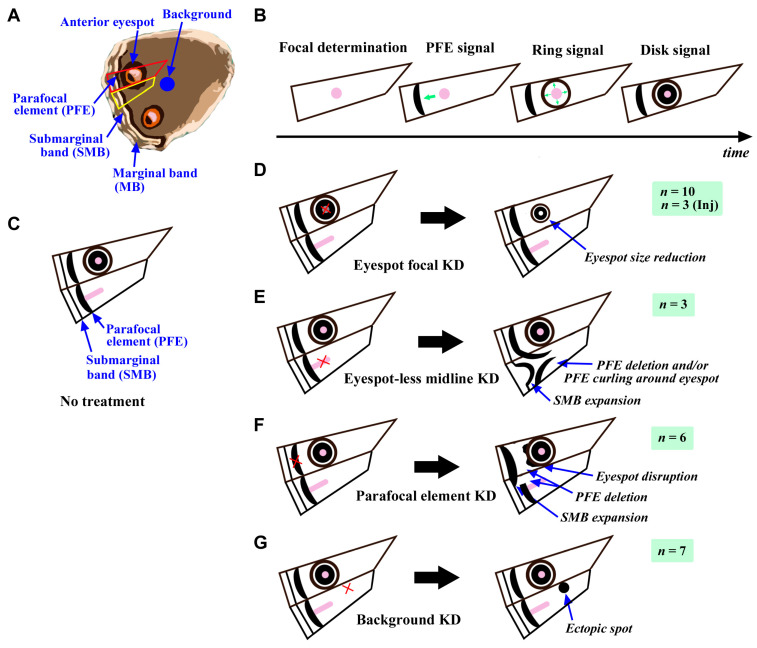
Interpretations of the antibody-mediated color pattern modifications in the present study. (**A**) An illustration of a hindwing with elemental nomenclature. The wing compartment indicated by red lines has both the anterior eyespot and parafocal element (PFE) in the adult wing. Their organizer is located at the prospective eyespot focus in the pupal wing tissue. The adjacent compartment indicated by the yellow lines does not have an eyespot but has a PFE, suggesting that the eyespot-less imaginary focal area is an organizing center for PFE. (**B**) Time series of color pattern determination proposed by the induction model. Green arrows indicate the directions of signal movement from the eyespot organizer (pink area). (**C**) Color pattern configuration of the normal wing (no treatment). Pink areas indicate Dll expression at the pupal stage. Additionally, the PFE and peri-wing veins may have their own Dll expression, but this is not shown in this illustration. (**D**–**G**) Interpretations of the knockdown results. The numbers of individuals that supported these interpretations via the sandwich method are indicated. For the sandwich method, the total number of individuals was 67 including both sexes. These individuals can be identified in Supplementary Materials. The crosses indicate the knockdown areas. (**D**) Eyespot focal knockdown (KD). In addition to the number of individuals via the sandwich method, the number of individuals supporting this interpretation via the injection method is indicated (Inj). For the injection method, the total number of individuals was 10 including both sexes. (**E**) Eyespot-less midline KD. (**F**) Parafocal element KD. (**G**) Background KD.

The present results may be interpreted as a sequence of color pattern determination proposed in the induction model [9,39,40], focusing on two compartments: one compartment including the anterior eyespot and another compartment without an eyespot (Figure 12A). Our previous color pattern analyses indicate that after the position of the eyespot focus is determined, the PFE signal is first released, followed by the ring signal and finally the disk signal (Figure 12B) [9,39,40]. If the organizer does not release ring and disk signals after the PFE signal is released, only PFE is formed without an eyespot in that compartment. We thus believe that the eyespot-less imaginary focus is the primary organizer for PFE, but its activity might have been ceased already at the pupal stage. Rather, the midline expression of Dll may be retained for PFE (Figure 12C).

The present protein knockdown experiment via the sandwich method resulted in a smaller eyespot, indicating the positive function of Dll in eyespot development (Figure 12D). In the sandwich method, this difference was statistically significant only for males (*p* = 0.0069), whereas for females, the *p*-value was close to the significance level (*p* = 0.083) (Figure 8). The anterior eyespots of females may be too large (thus, the Dll expression level may be too high) to repress its function via the delivered antibody molecules. A reduction in eyespot size was also observed with the injection method. This reduction in eyespot size is consistent with the previous understanding that Dll is a positive regulator of eyespot size [16,30,31,32].

Additionally, the PFE in the eyespot-less compartment invaginated toward the imaginary focus or curved along the adjacent eyespot (Figure 12E). In this type of modification, a PFE signal did not proceed toward the distal direction and was positioned around an adjacent eyespot. This invagination or pulling may be similar to the “drawing effect” observed in a damage study [27]. This PFE invagination may be caused by the midline or peri-wing vein Dll knockdown. In some individuals, PFE invagination was observed toward the eyespot focus in an eyespot-existing compartment (Figure 9M–P). This type of modification may be induced by the partial eyespot focal inhibition and may be mechanistically similar to the midline knockdown. These results suggest that a PFE is dependent on the neighboring eyespots, midline, and/or peri-wing veins for its position and shape. This is consistent with previous color pattern analyses [4,9] and a PFE-inclusive mathematical model [1,41] in that both the eyespot and PFE may belong to the border symmetry system. The curved PFE is reminiscent of some results in the source and sink model [1] and in the recent grass fire model [41].

Furthermore, the submarginal band (SMB) expanded toward the basal side in conjunction with the PFE invagination (Figure 12E), probably because of the lack of elements in this area due to the PFE invagination. Repulsive interactions among elements have been proposed experimentally [26,27] but have often been dismissed. This was observed here as an expansion of SMB in the compartment of the PFE invagination.

There is a case where Dll in the secondary PFE organizer in the prospective PFE focus appeared to be inhibited by the antibody (Figure 9K,L). In this case, PFE was likely deleted (Figure 12F). The outer ring of the eyespot was disrupted with possible fusion with the remaining PFE in the same compartment. The SMB was expanded, probably due to the vacancy of the PFE-occupying area. This case can be interpreted as the demonstration of the secondary PFE organizer and the repulsive interactions among elements. The serial induction of organizers on the wing epithelium is proposed to be an important concept in butterfly wing color pattern development, resulting in the fractal geometry of color pattern elements [6]. Interestingly, previous studies revealed that peripheral bands including PFEs were disrupted but not eyespots by the WntA or Fz2 knockout [42,43,44]. The PFE may have its own organizing center that operates differently from an eyespot organizing center.

Furthermore, Dll inhibition in the background area resulted in the emergence of black spots (Figure 12G). The low-level Dll expression observed widely in the background area [11,20,21] has been enigmatic. Its Dll expression may have an inhibitory effect on preventing ectopic spots from emerging. This response is clearer in males than in females. Additionally, the anti-Dll antibody treatment appeared to affect the scale development. Thus, Dll may also function to assist scale development throughout wings. This result is also consistent with a previous study [31]. However, we could not clearly detect the Dll expression in the background area except for the peri-wing veins. Expression studies should be performed thoroughly in the future.

These results generally demonstrate the function of Dll in establishing a developmental organizer for eyespots and PFEs. How does Dll specify eyespots and PFEs? A molecular model has been proposed by Connahs et al. (2019) [31], but regulatory molecules should ultimately be linked to the fate determination and phenotypic expression of scale cells. We speculate that Dll expression may induce mechanical morphogen, a fate-determining signal [45]. This signal may induce mechanical changes in the cytoskeletal machinery of a scale cell similar to a cell state splitter [46,47,48,49].

Additionally, there are still several enigmas regarding the Dll expression and its phenotype. First, eyespot size is affected not only by the Dll expression but also by other factors [18,19]. Such factors may be genetic, hormonal, or environmental. Similarly, Dll alone may not be able to induce a full eyespot [19,32]. This may be partly because an eyespot is a complex (but not a simple) element with multiple subelements. Second, PFE cannot be eliminated by physical damage at the imaginary focus, but color pattern modifiers such as tungstate [50], heparin [51], and FB28 [52] can modify the PFE signal after pupation. An explanation for these findings is that the PEF signal has already been released by the time of physical damage. However, Dll knockdown appears to cancel or change the PFE signal. We speculate that even after the release of the PFE signal, the midline or the functional imaginary focus is required to establish the secondary PFE organizer at the right place. Physical damage may inactivate the imaginary focus but simultaneously activate ectopic activity, as in background damage.

## 5. Conclusions

The present study demonstrated the effectiveness of an antibody-mediated protein delivery method for understanding the function of Dll in vivo in butterfly wing color pattern development. Although the precise molecular functions of Dll depend on the downstream genes that are expressed after Dll activation, Dll appears to function to establish an organizer for the eyespot. Dll also functions to establish the primary and secondary organizers for PFE, suggesting that both the eyespot and PFE belong to the border symmetry system. We believe that Dll is important for coordinating the border symmetry system. Dll may also function to inhibit the emergence of ectopic elements in the background area and to support scale development. One study on *Drosophila* suggests that Dll affects cell adhesion to make a morphological bump three-dimensionally [14]. This may be consistent with the fact that the eyespot organizer is three-dimensional with pupal cuticle focal spots and marks [53,54].

## Figures and Tables

**Table 1:** Survival rate and modification rate of the treated individuals via the sandwich method *.

Antibody	Treated	Eclosion Success	Patterns Modified	Eclosion Failure	Pupal Death	Survival Rate (SR)	Modification Rate (MR)
Anti-Dll antibody	78	67	23	8	3	85.9%	34.3%
Anti-spike antibody	74	63	7	7	4	85.1%	11.1%

* The numbers of individuals are shown in this table. Individuals with eyespot size reduction are not included.

**Table 2:** Survival rate and modification rate of the treated individuals via the injection method *.

Antibody	Treated	Eclosion Success	Patterns Modified	Eclosion Failure	Pupal Death	Survival Rate (SR)	Modification Rate (MR)
Anti-Dll antibody	12	10	3	0	2	83.3%	30.0%
Anti-spike antibody	16	15	0	1	0	93.7%	0%

* The numbers of individuals are shown in this table. Individuals with eyespot size reduction and impaired scales are included.

## Data Availability

This paper and Supplementary Materials contain all relevant data obtained in this study.

## Supplementary Materials

The following supporting information can be downloaded at: https://www.mdpi.com/article/10.3390/cells13171476/s1, Supplementary File S1: Anti-Dll antibody sandwich female; Supplementary File S2: Anti-Dll antibody sandwich male; Supplementary File S3: Anti-spike antibody sandwich female; Supplementary File S4: Anti-spike antibody sandwich male; Supplementary File S5: Anti-Dll antibody injection both sexes; Supplementary File S6: Anti-spike antibody injection both sexes.

## Appendix A

Both the anti-Dll antibody and the anti-spike antibody that were produced in this study were purified via an affinity column, and their titers were evaluated via ELISA as shown in Figure A1.
