# Supplementary material for: Antibody-Mediated Protein Knockdown Reveals Distal-less Functions for Eyespots and Parafocal Elements in Butterfly Wing Color Pattern Development
Source: Cells. 2024 Sep 2;13(17):1476. doi: 10.3390/cells13171476 (PMC11394314; doi:10.3390/cells13171476)
Supplement: Supplementary file 1 [file cells-13-01476-s001.zip › Supplementary file 6, Anti-spike antibody injection both sexes.pdf]

Supplementary file 6

Anti-spike antibody, injection method, both sexes

Female

NO1

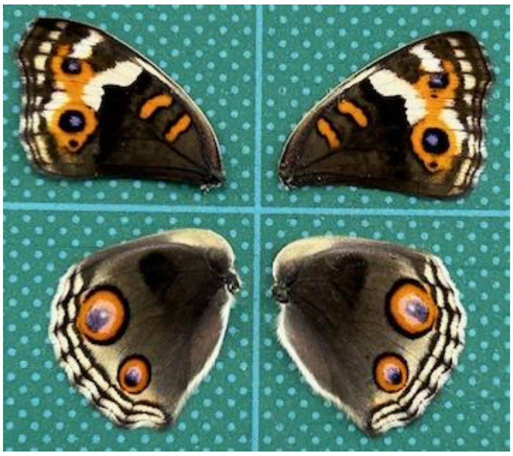

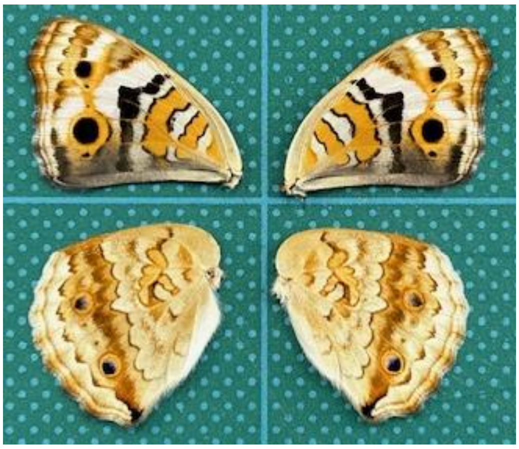

NO2

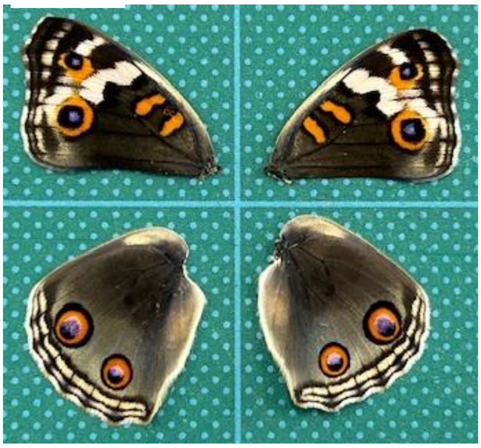

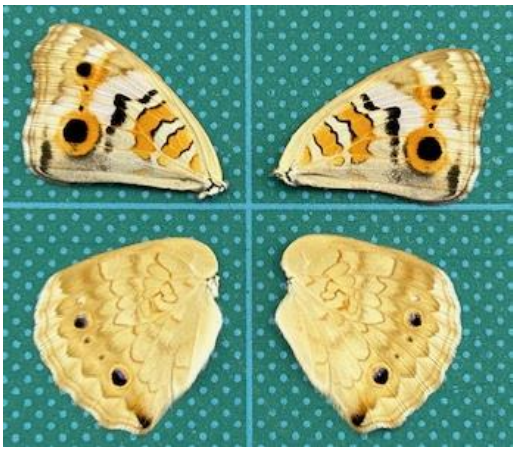

NO3

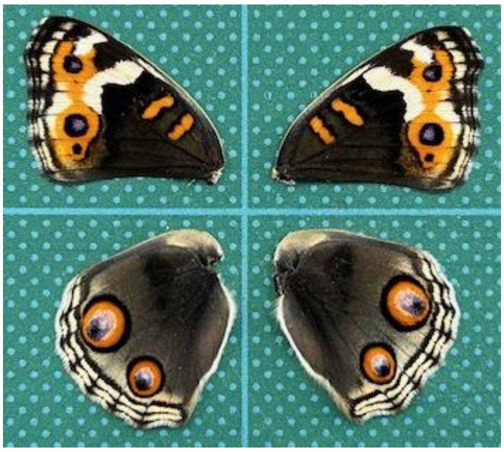

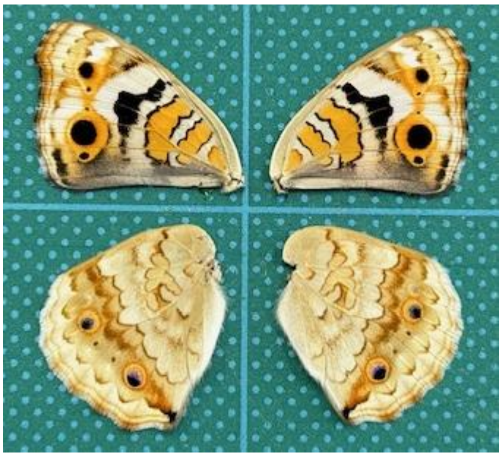

NO4

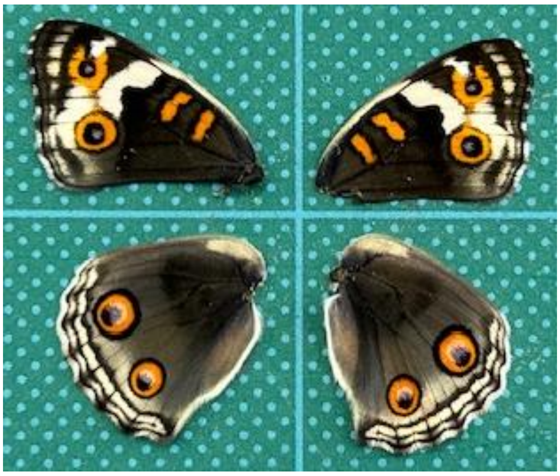
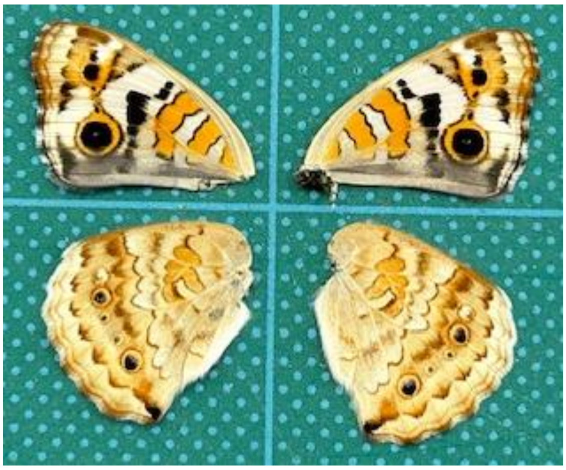

NO5

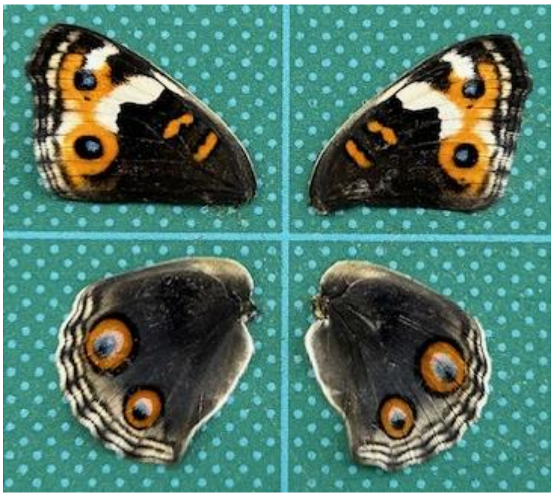
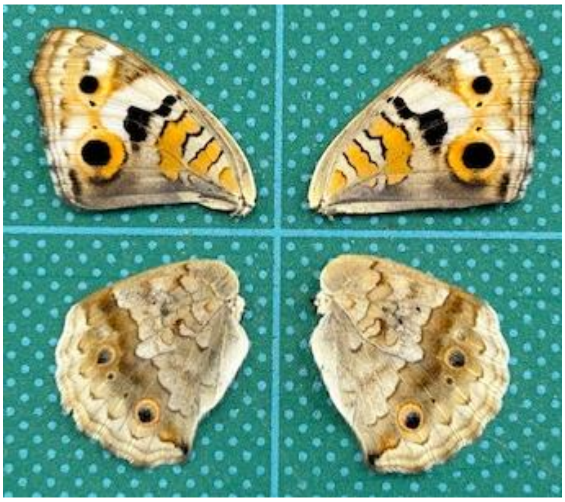

NO6

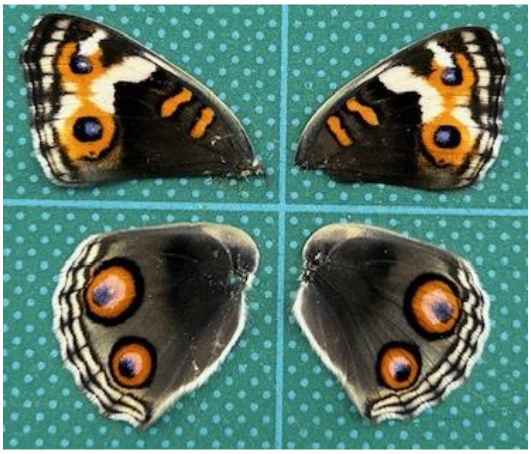
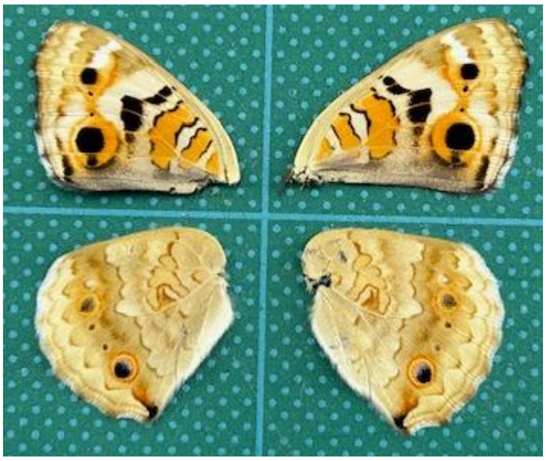

NO7

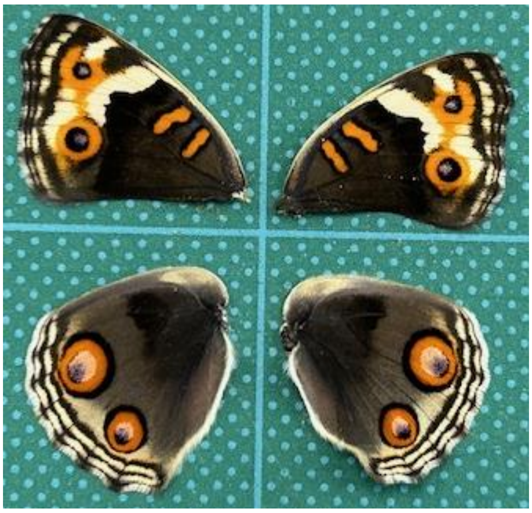 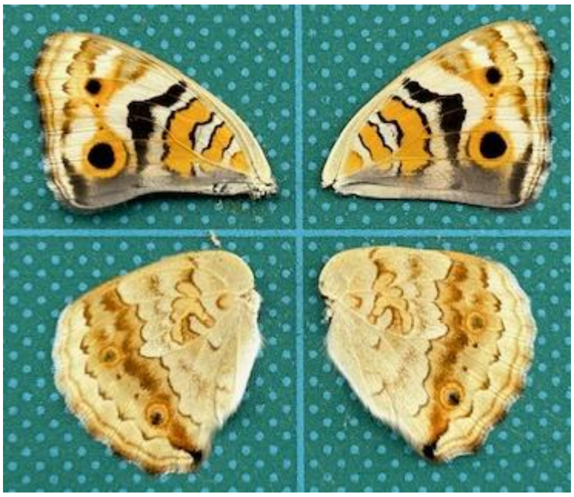

NO8

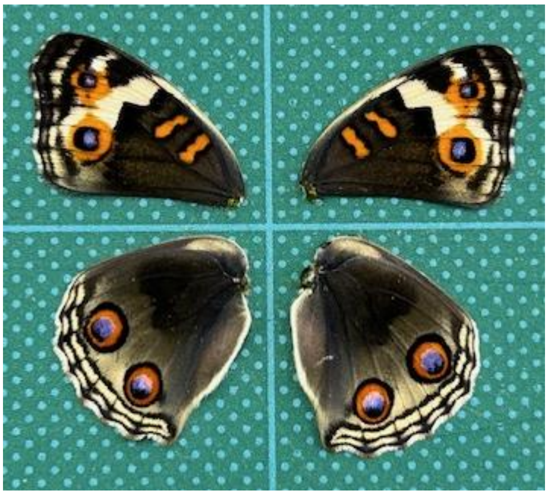 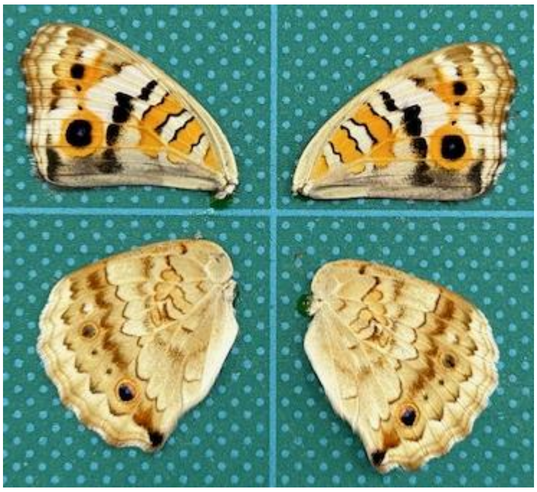

Male

NO1

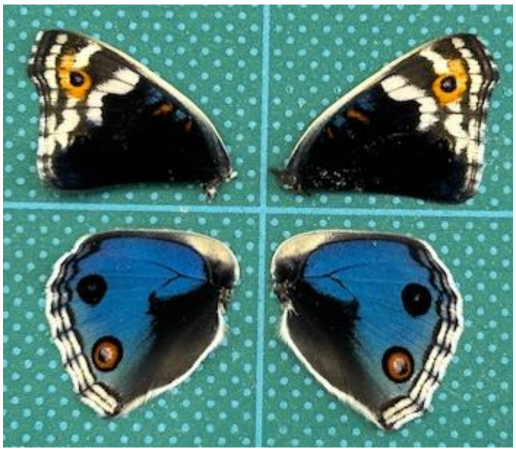 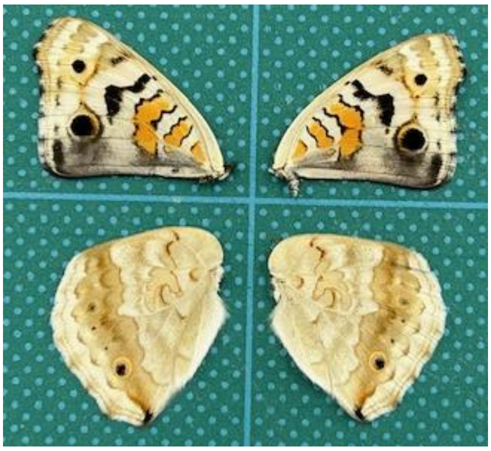

NO2

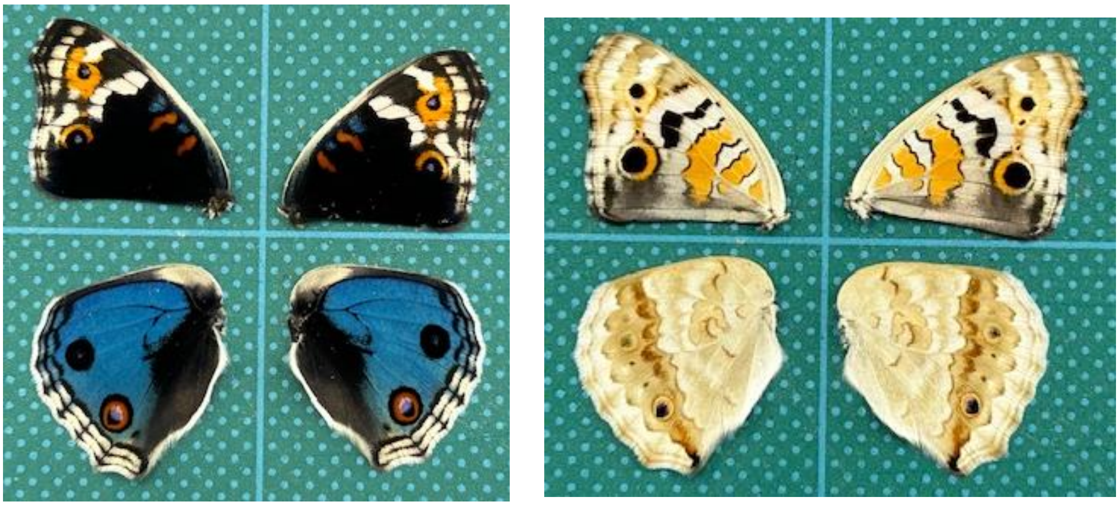

NO3

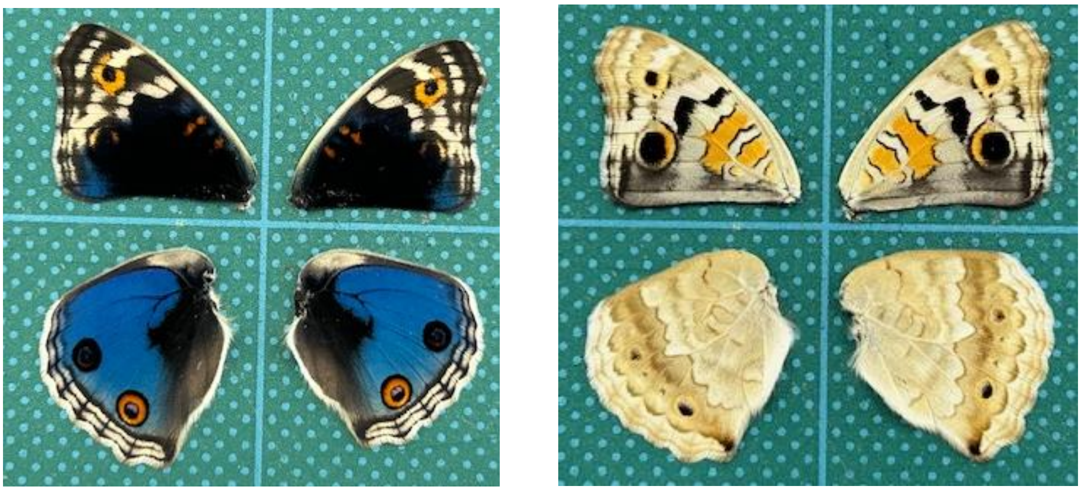

NO4

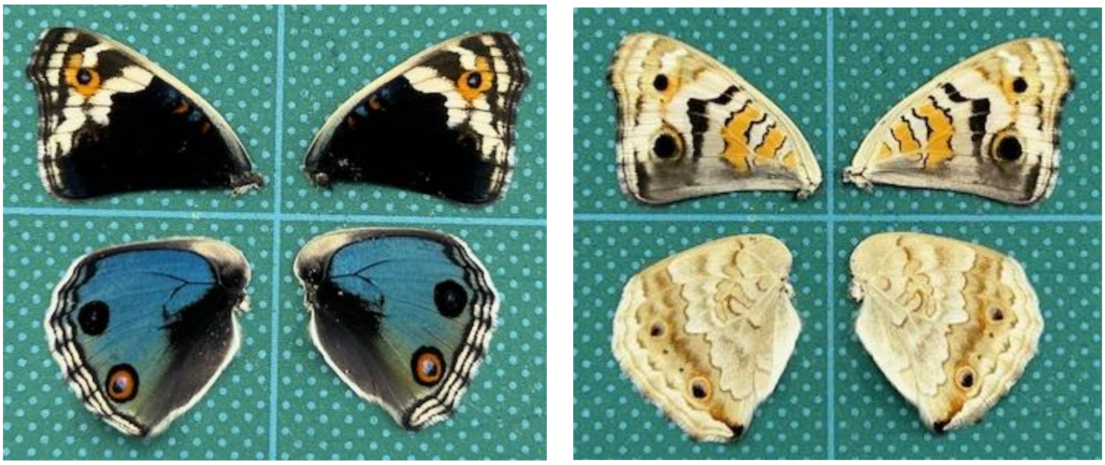

NO5

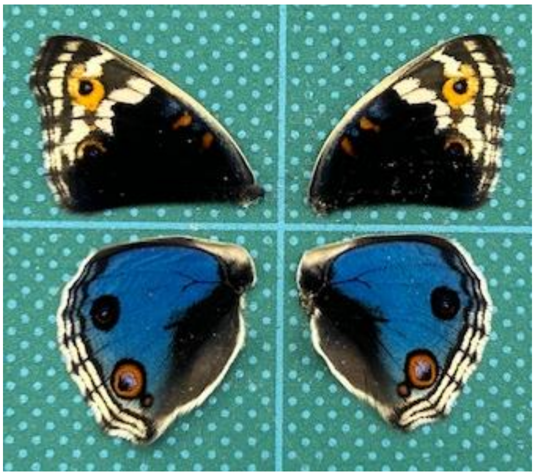
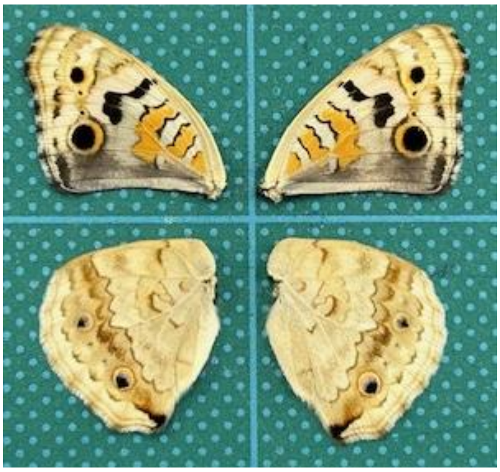

NO6

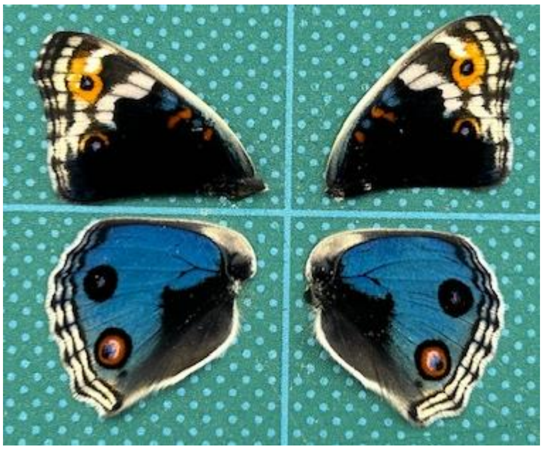
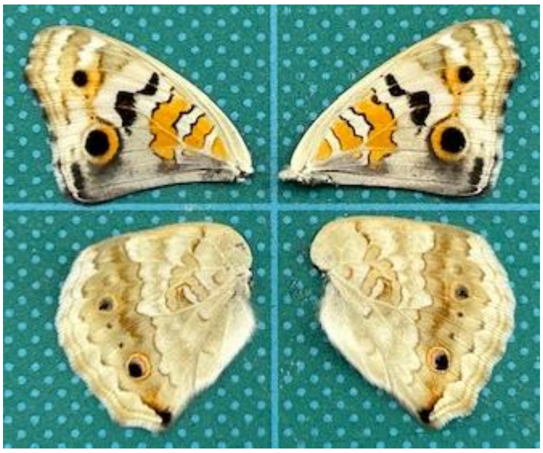

NO7

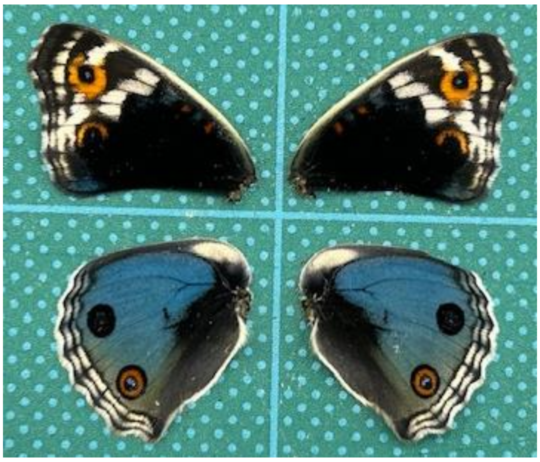
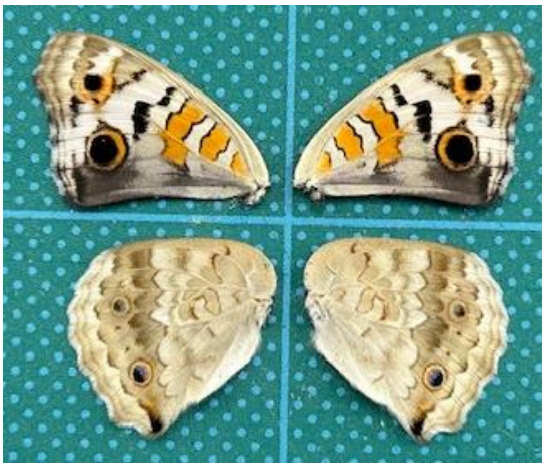

Summary: Anti-spike antibody, Injection, Both sexes

TOTAL number of successful eclosion = 15

TOTAL number of modified individuals (including size change) = 0

Note: In these images, the distance between the centers of adjacent dots is 1.5 mm.
