# Supplementary material for: Antibody-Mediated Protein Knockdown Reveals Distal-less Functions for Eyespots and Parafocal Elements in Butterfly Wing Color Pattern Development
Source: Cells. 2024 Sep 2;13(17):1476. doi: 10.3390/cells13171476 (PMC11394314; doi:10.3390/cells13171476)
Supplement: Supplementary file 1 [file cells-13-01476-s001.zip › Supplementary file 5, Anti-Dll antibody injection both sexes.pdf]

Supplementary file 5

Anti-Dll antibody, injection method, both sexes

Female

NO1

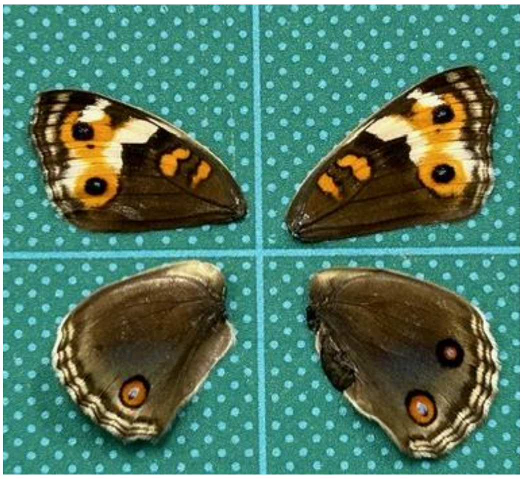
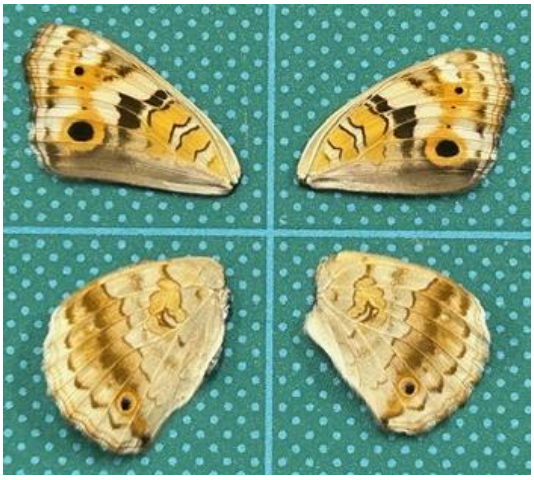

NO2

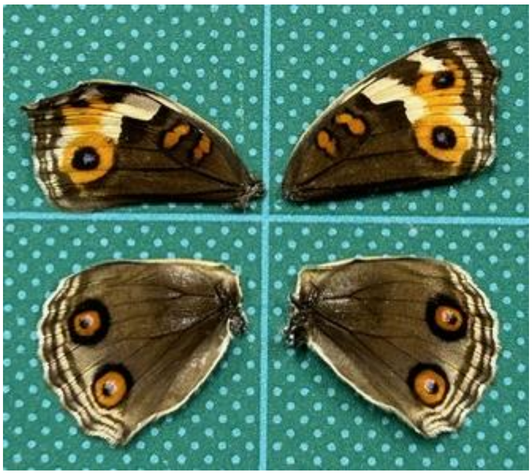
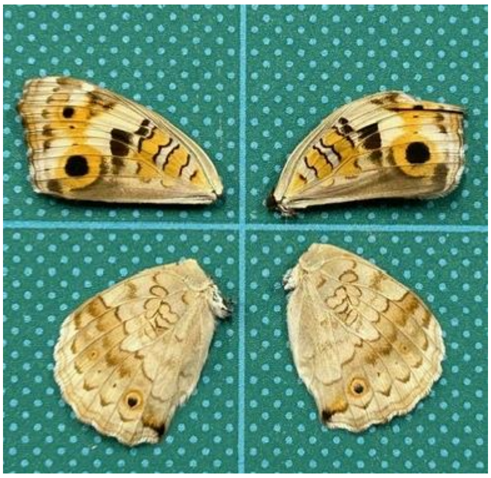

NO3

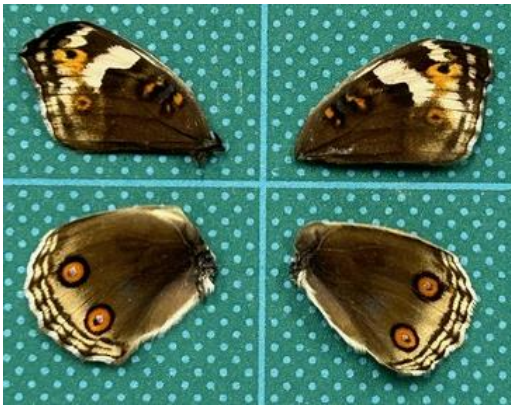
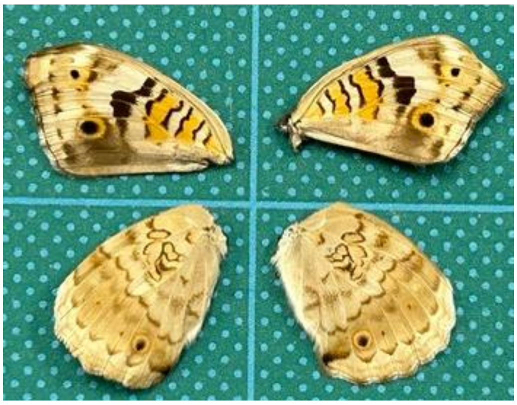

NO4

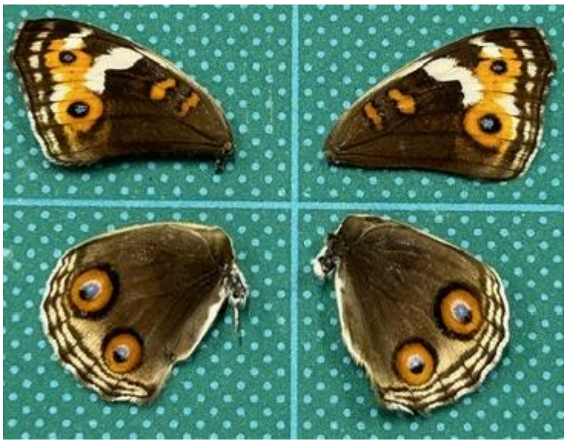

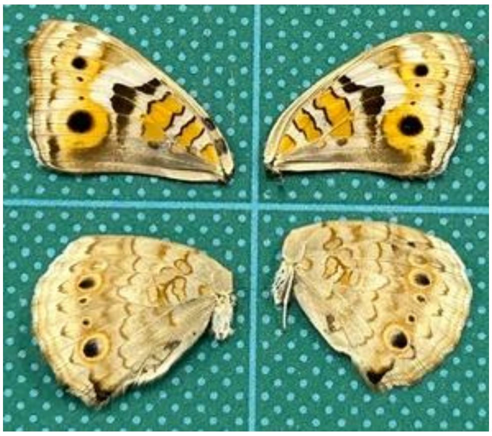

NO5

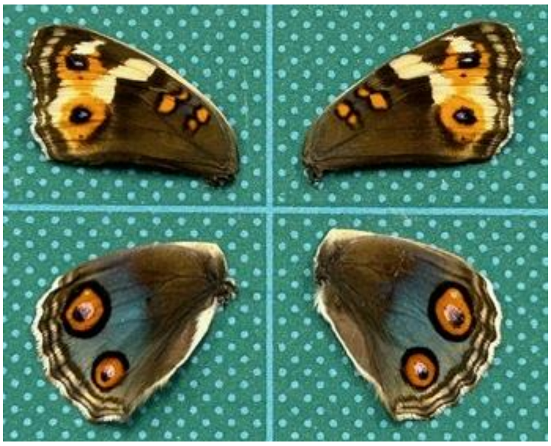

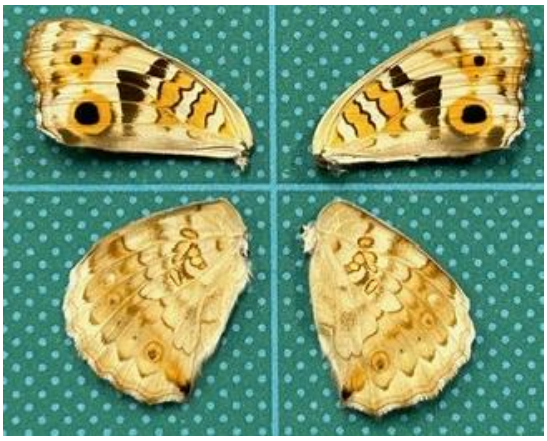

NO6

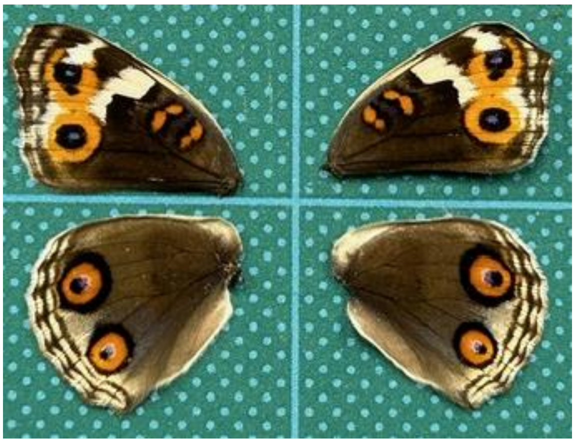

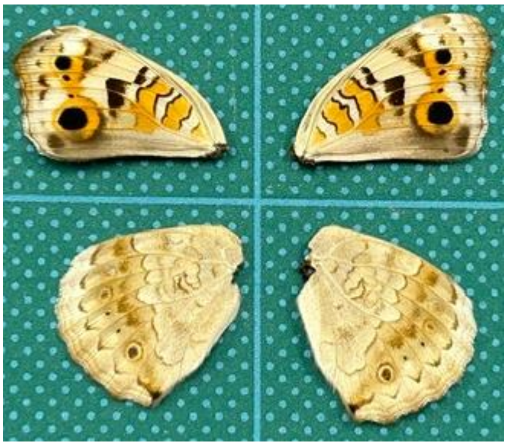

NO7

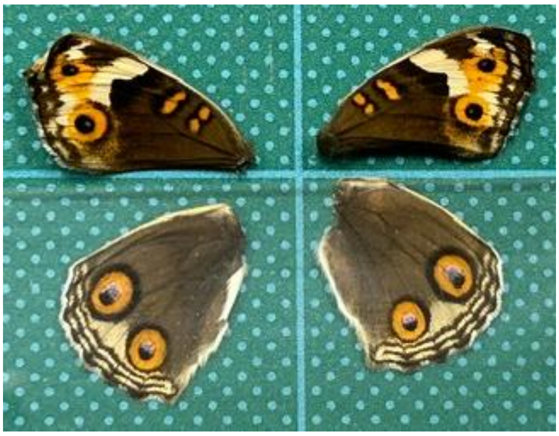 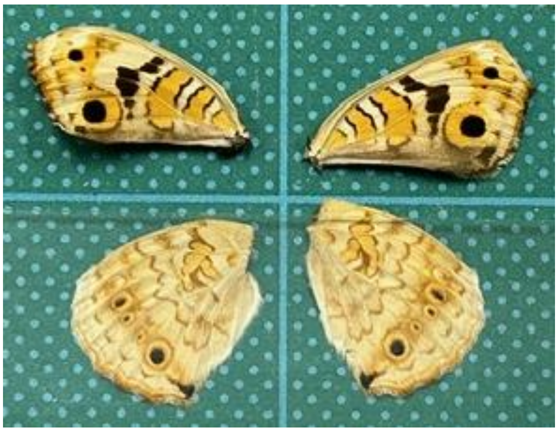

Male

NO1

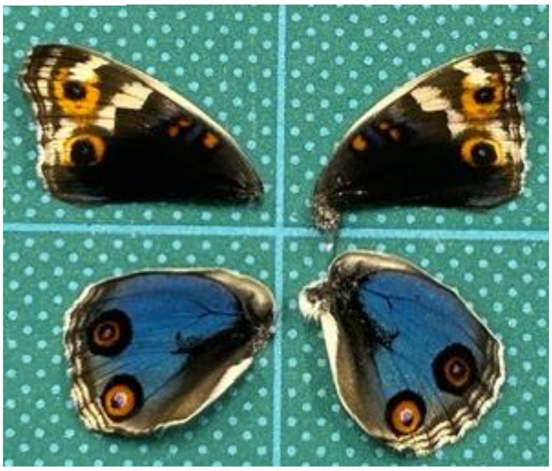 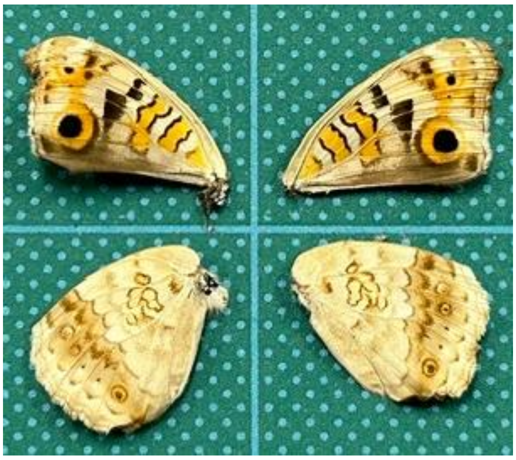

NO2

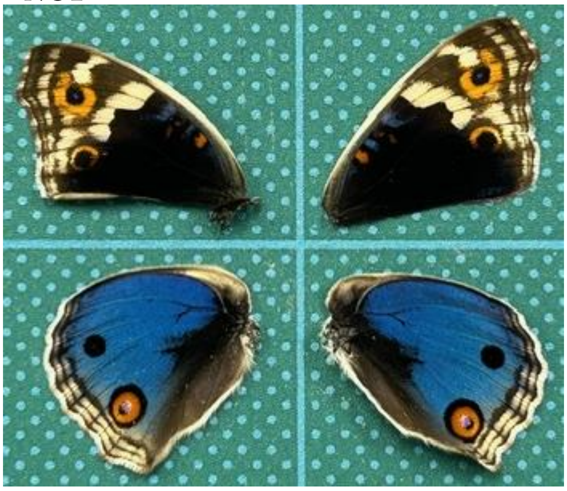 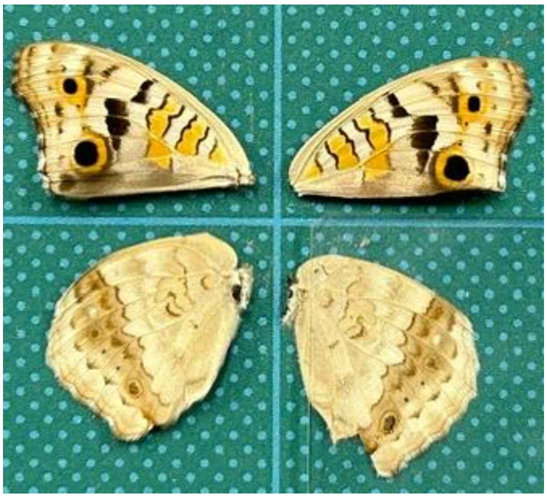

NO3

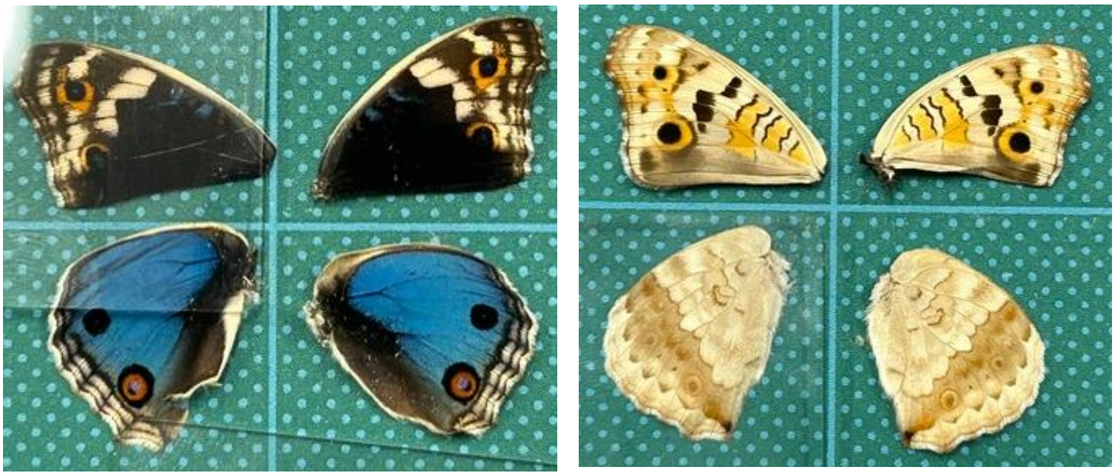

Summary: Anti-Dll antibody, Injection, Both sexes

<u>TOTAL number of successful eclosion = 10</u>

Eyespot size reduction:

No.1, No.3 (*n* = 2)

Impaired scale development:

No.1 (redundant), No.2 (*n* = 2)

<u>TOTAL number of modified individuals (including size change) = 3</u>

Note: In these images, the distance between the centers of adjacent dots is 1.5 mm.
