# Supplementary material for: Antibody-Mediated Protein Knockdown Reveals Distal-less Functions for Eyespots and Parafocal Elements in Butterfly Wing Color Pattern Development
Source: Cells. 2024 Sep 2;13(17):1476. doi: 10.3390/cells13171476 (PMC11394314; doi:10.3390/cells13171476)
Supplement: Supplementary file 1 [file cells-13-01476-s001.zip › Supplementary file 2, Anti-Dll antibody sandwitch male.pdf]

Supplementary file 2

Anti-Dll antibody, sandwich method, male

Male

NO1

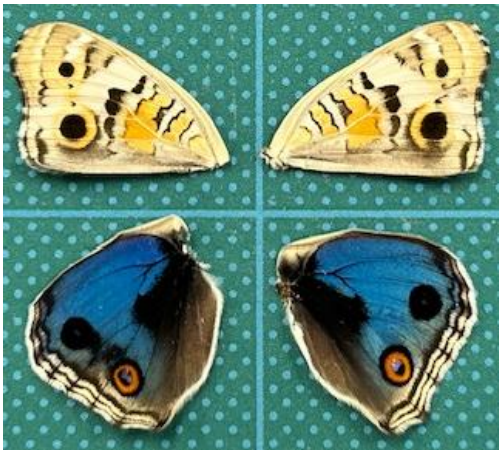

NO2

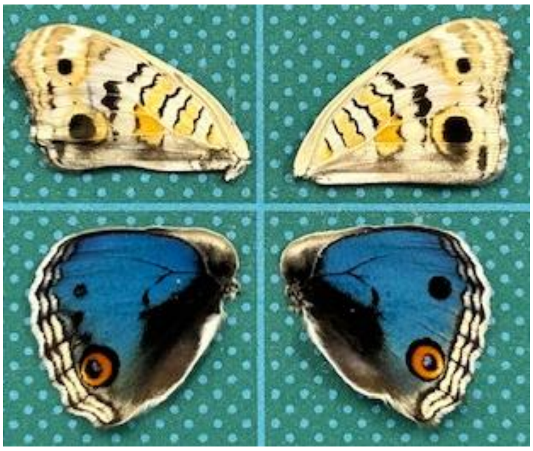

NO3

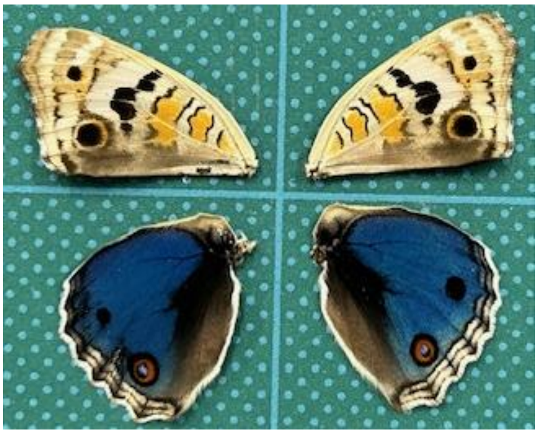

NO4

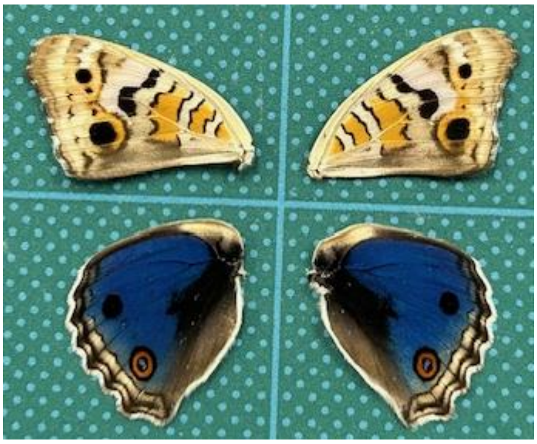

NO5

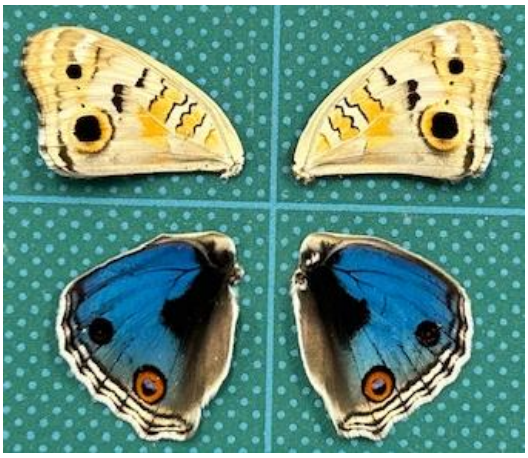

NO6

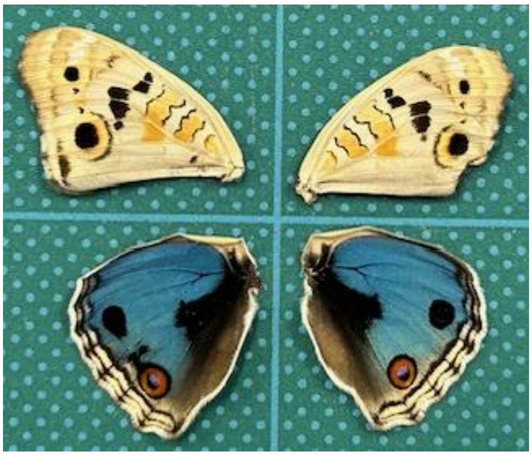

NO7

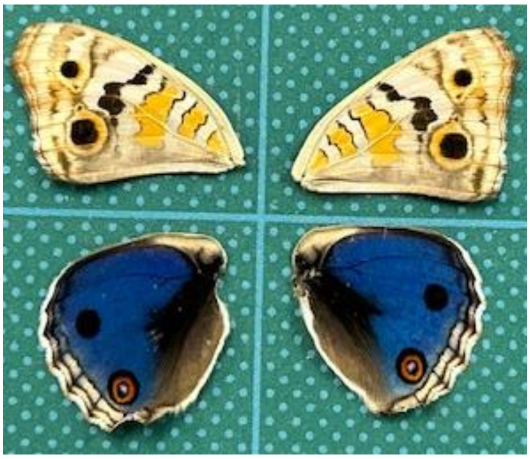

NO8

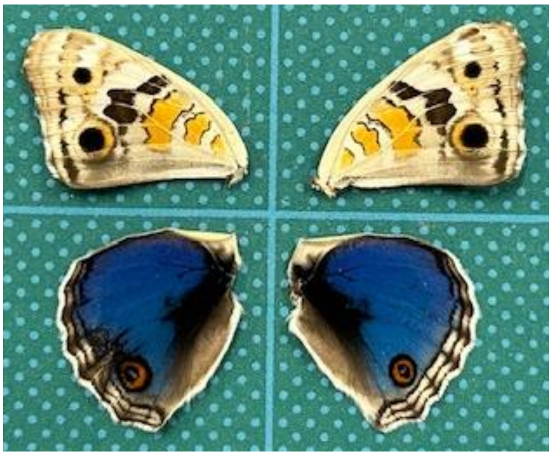

NO9

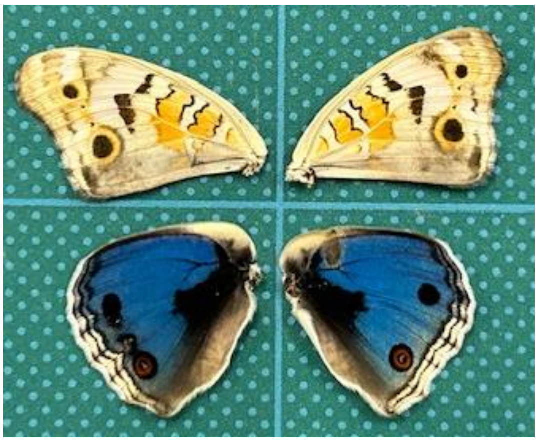

NO10

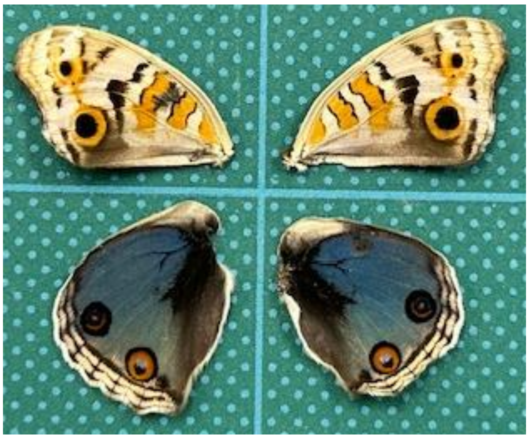

NO11

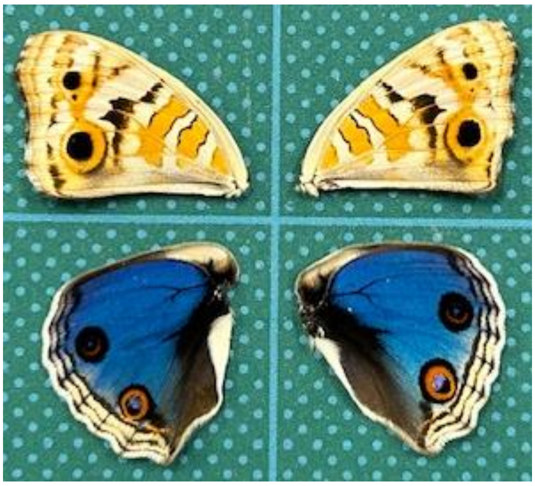

NO12

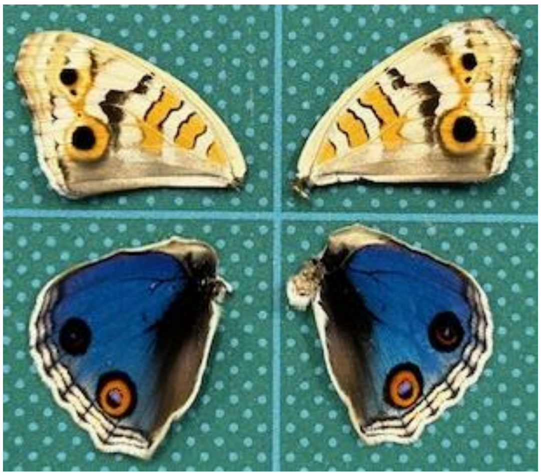

NO13

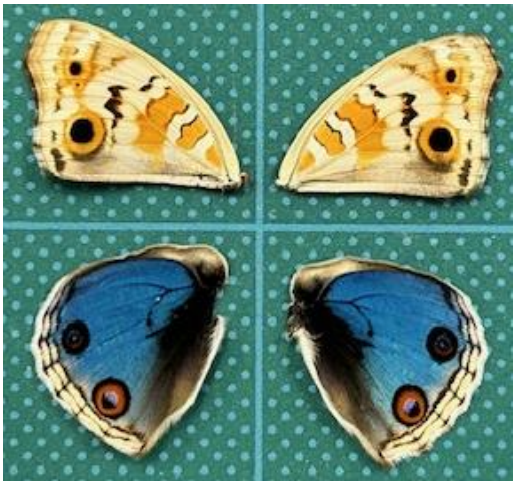

NO14

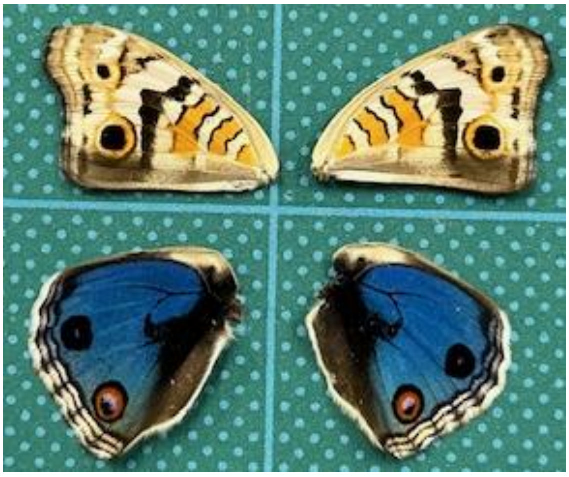

NO15

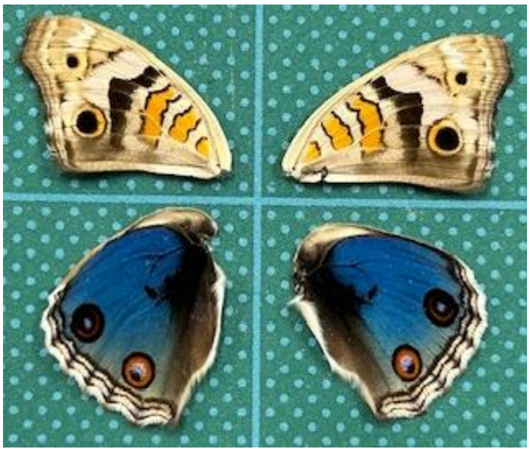

NO16

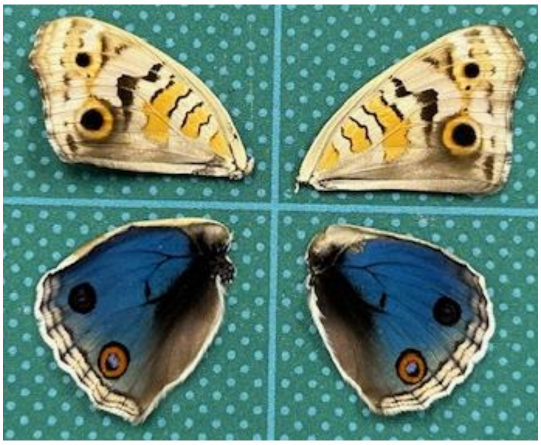

NO17

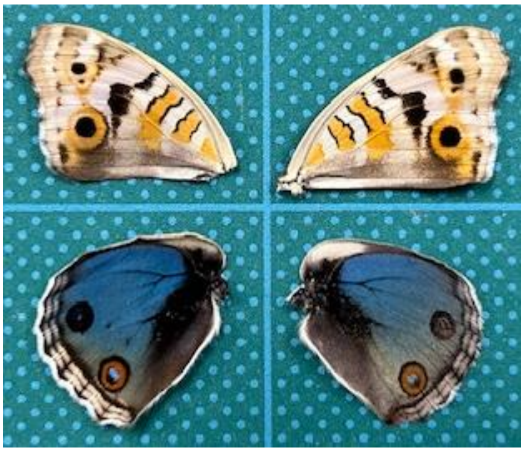

NO18

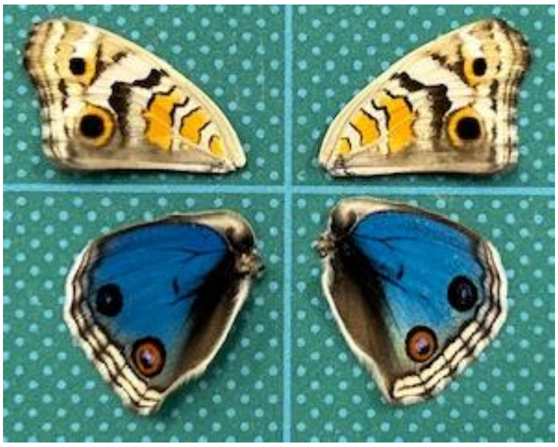

NO19

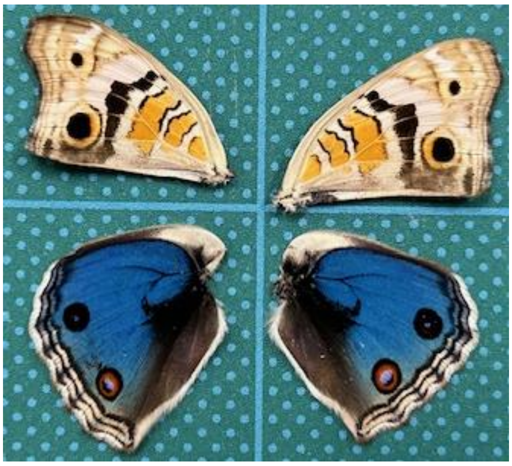

NO20

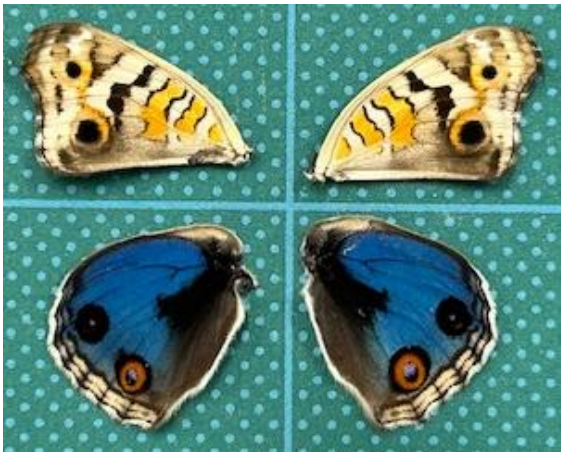

NO21

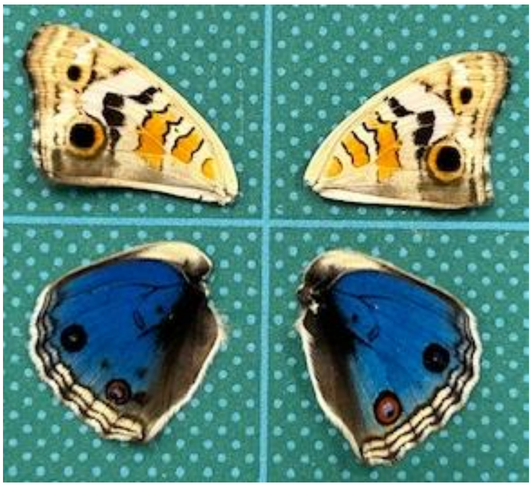

NO22

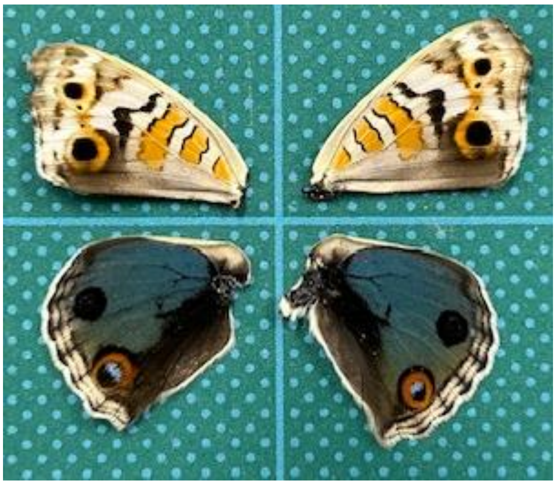

NO23

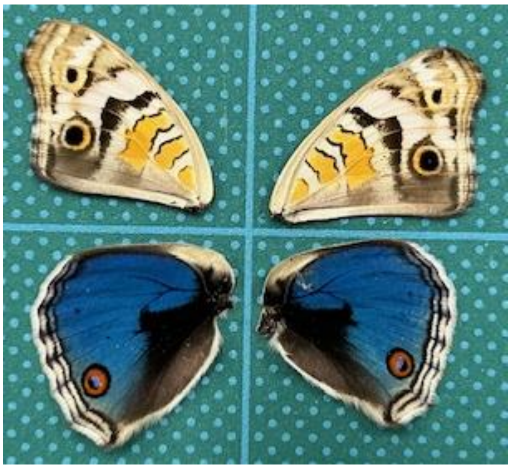

Summary: Anti-Dll antibody, Sandwich, Male

TOTAL number of successful eclosion = 23

Black spot and partial PFE pulling (invagination):

No.1, No.3, No.6, No.7, No.8, No.9 ($n = 6$)

TOTAL number of modified individuals (excluding size change) = 6

Note: In these images, the distance between the centers of adjacent dots is 1.5 mm.
