# Supplementary material for: Antibody-Mediated Protein Knockdown Reveals Distal-less Functions for Eyespots and Parafocal Elements in Butterfly Wing Color Pattern Development
Source: Cells. 2024 Sep 2;13(17):1476. doi: 10.3390/cells13171476 (PMC11394314; doi:10.3390/cells13171476)
Supplement: Supplementary file 1 [file cells-13-01476-s001.zip › Supplementary file 1, Anti-Dll antibody sandwitch female.pdf]

Supplementary file 1

Anti-Dll antibody, sandwich method, female

Female

NO1

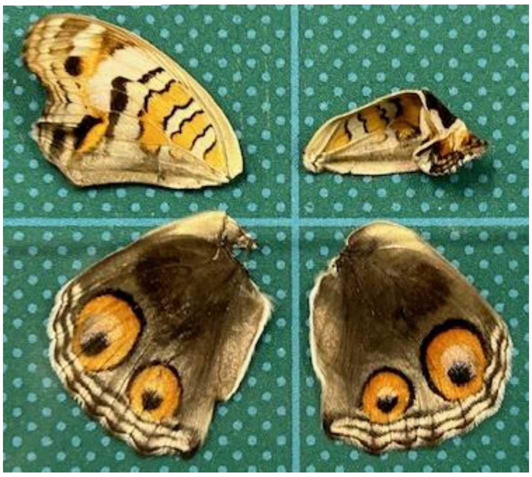

NO2

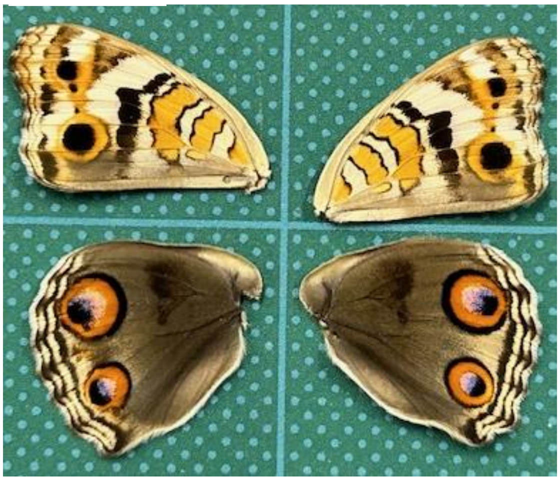

NO3

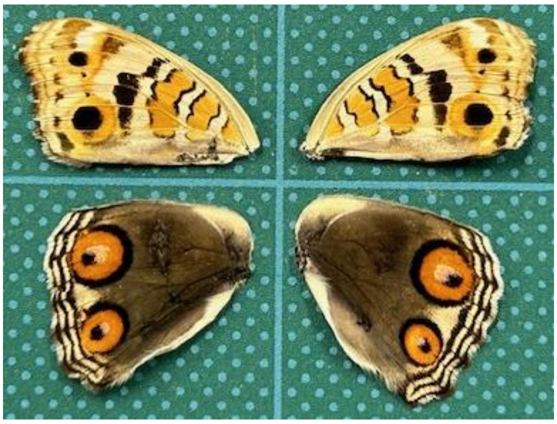

NO4

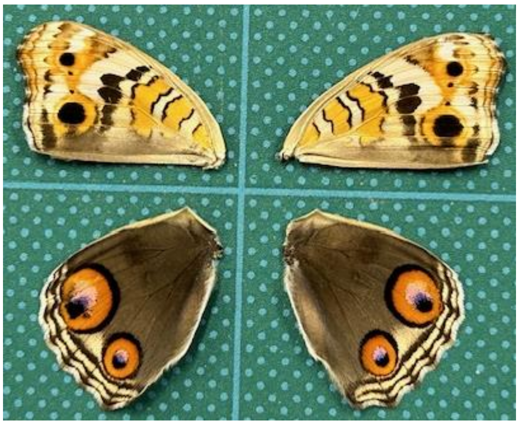

NO5

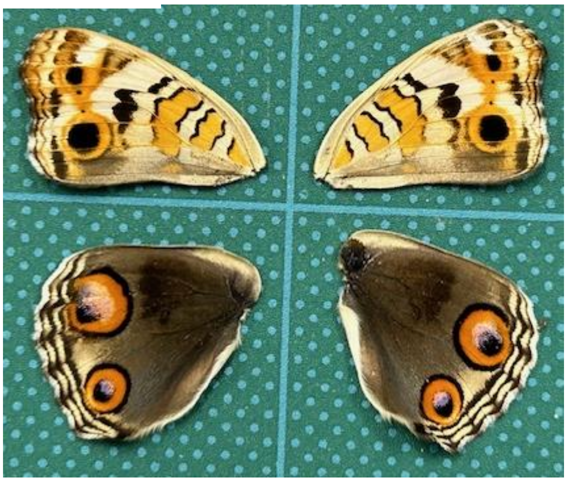

NO6

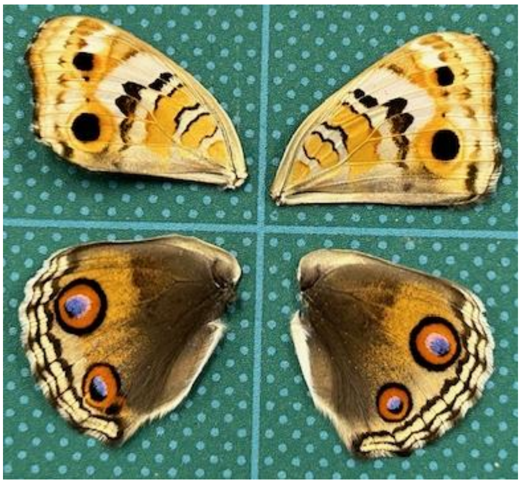

NO7

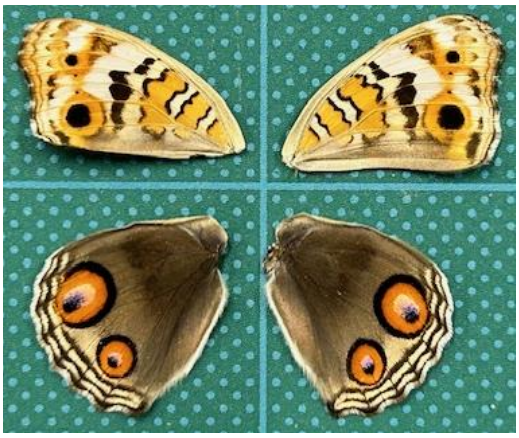

NO8

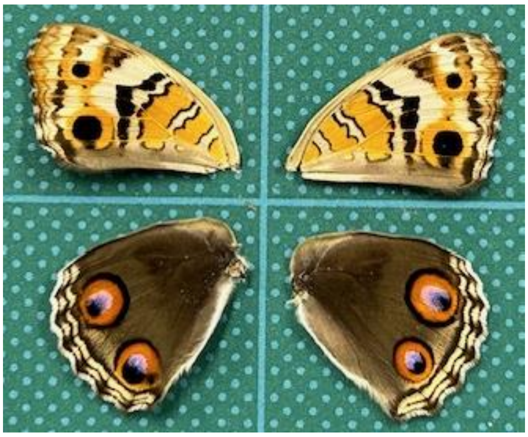

NO9

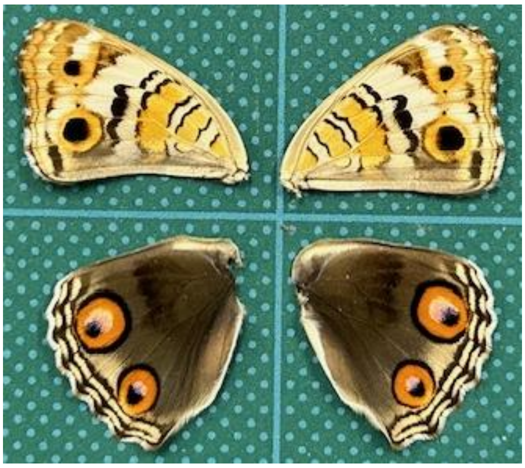

NO10

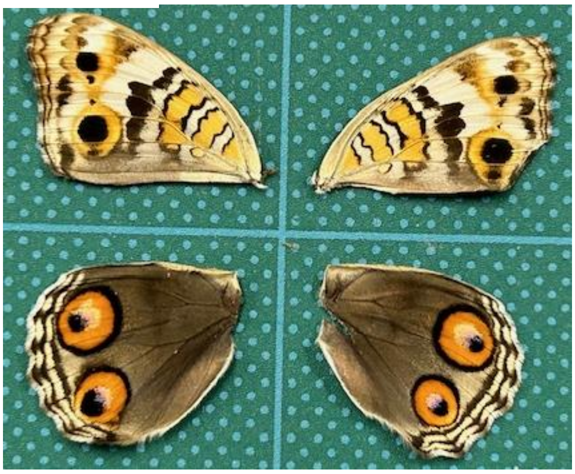

NO11

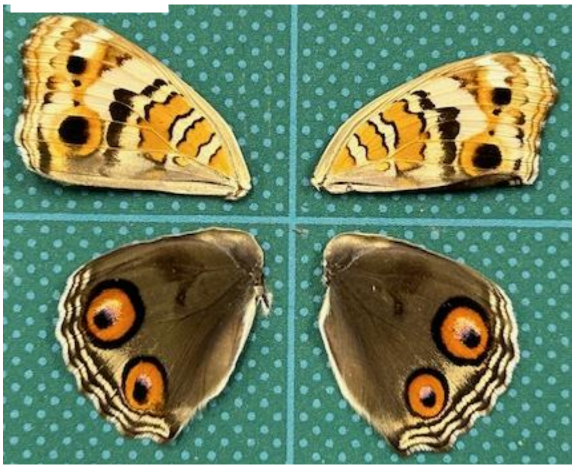

NO12

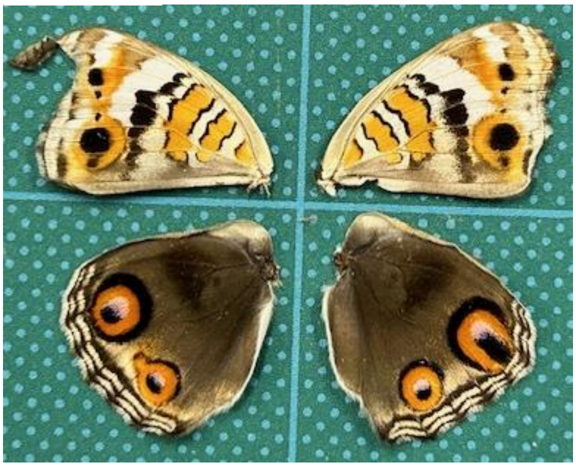

NO13

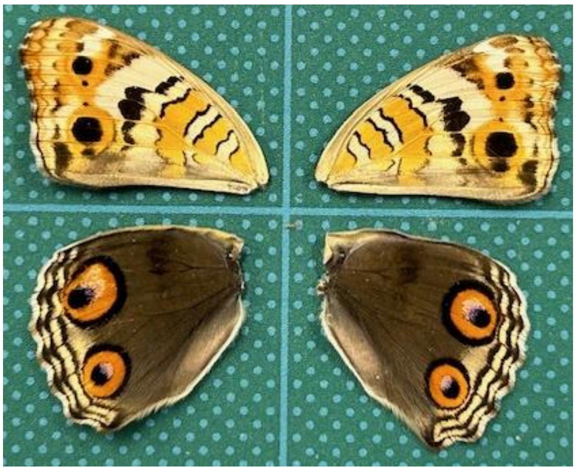

NO14

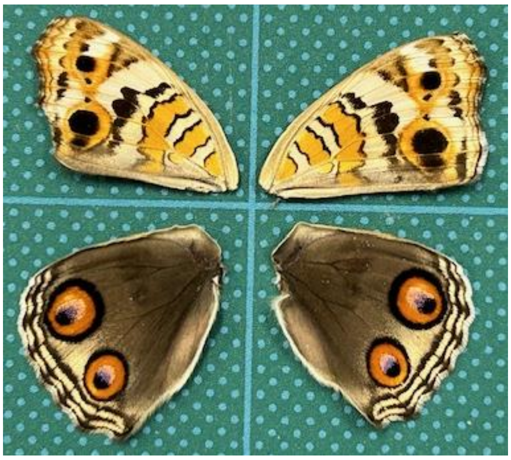

NO15

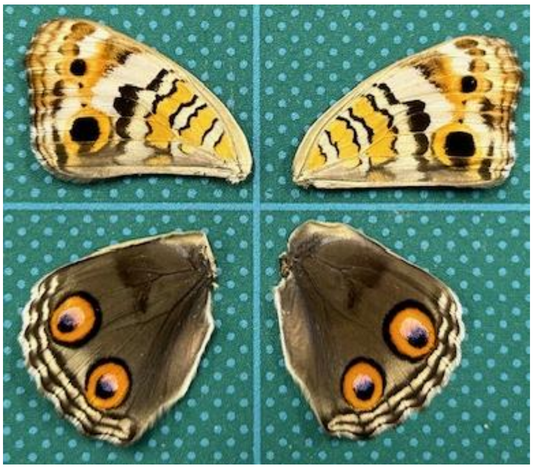

NO16

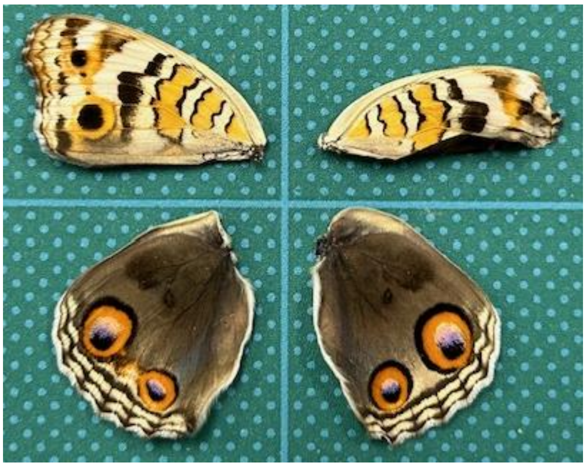

NO17

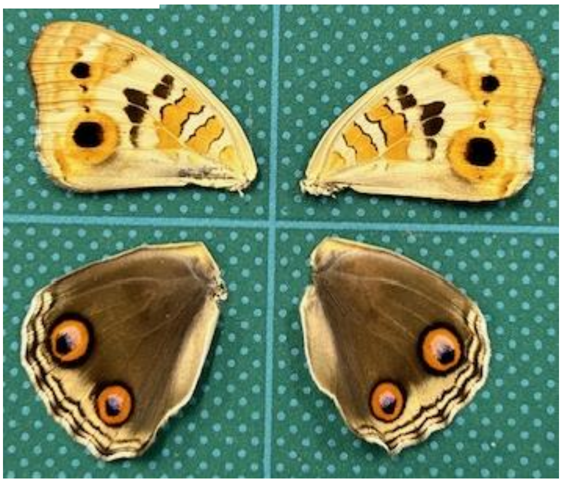

NO18

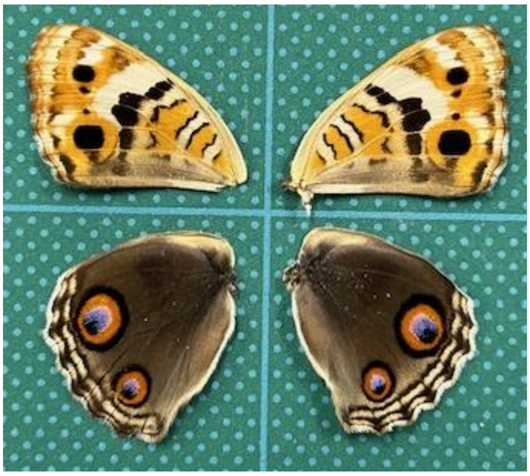

NO19

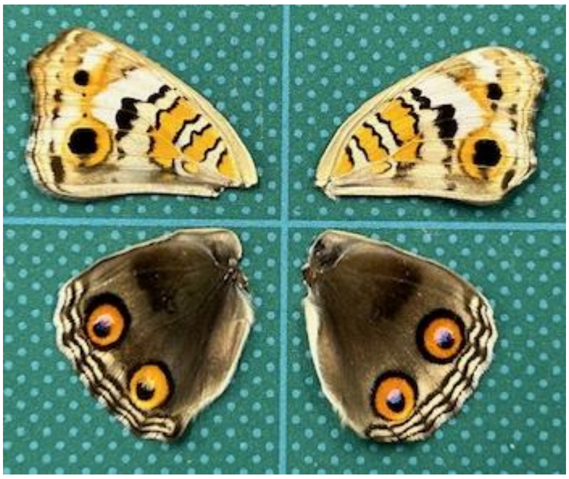

NO20

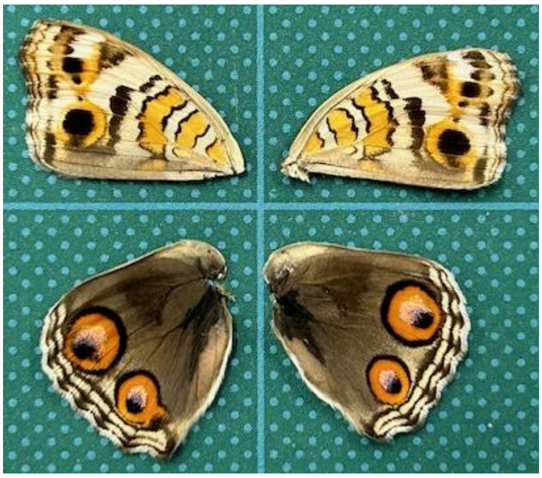

NO21

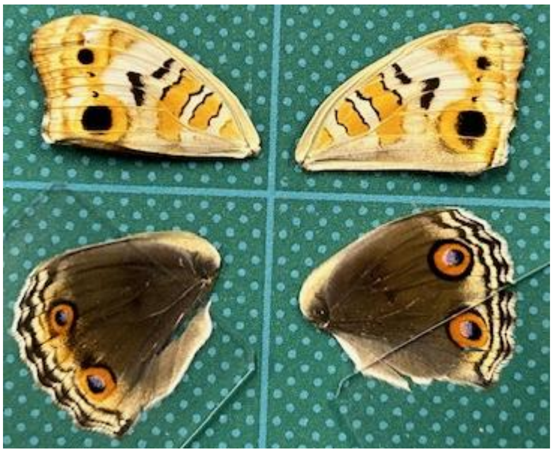

NO22

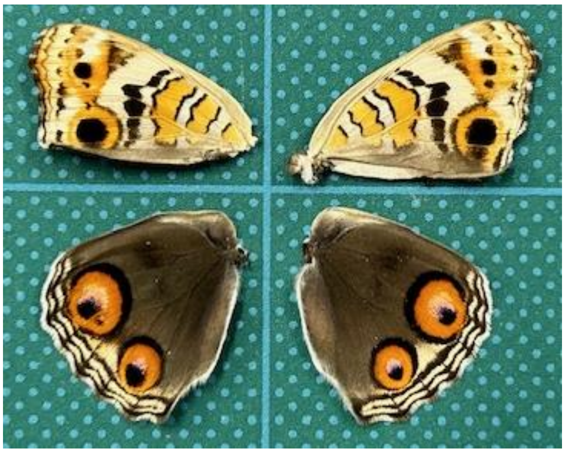

NO23

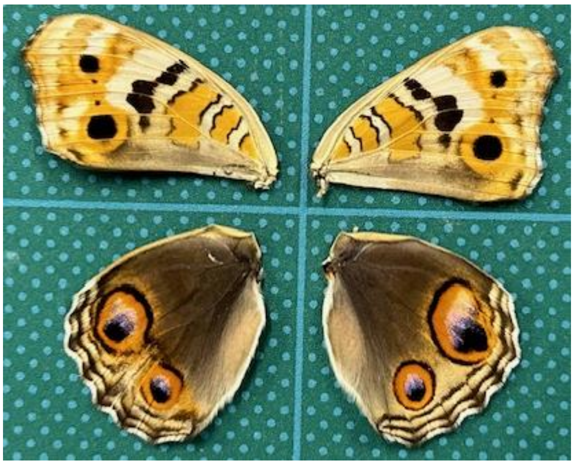

NO24

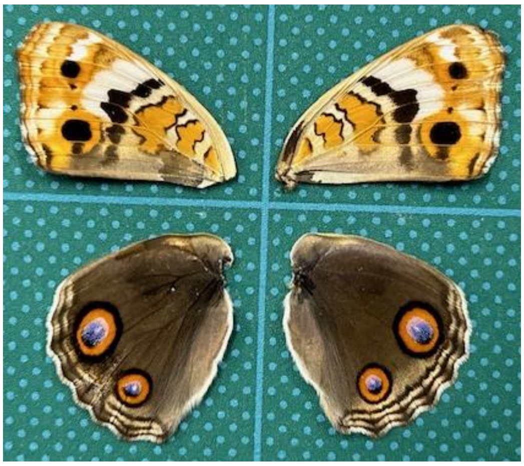

NO25

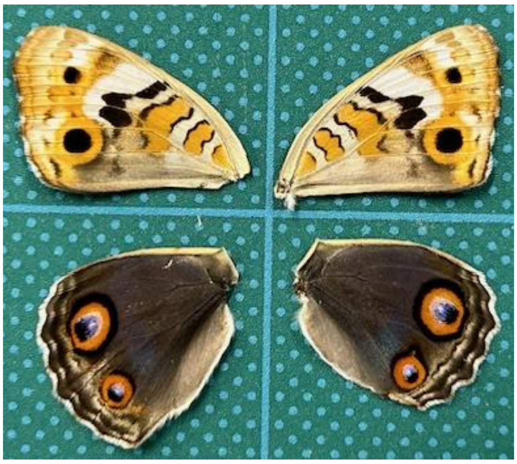

NO26

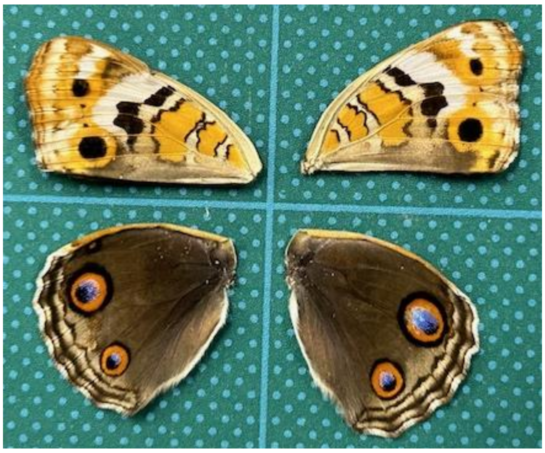

NO27

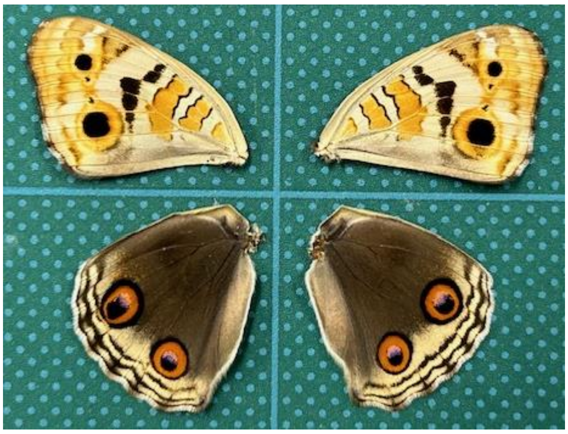

NO28

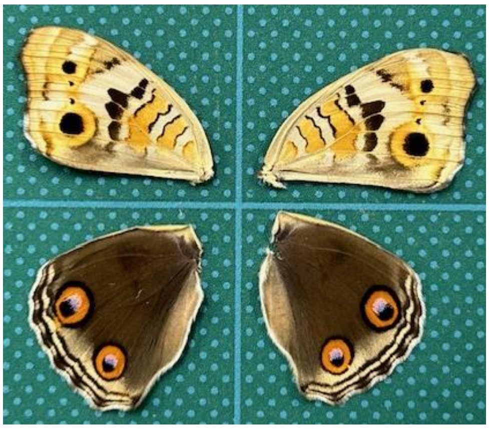

NO29

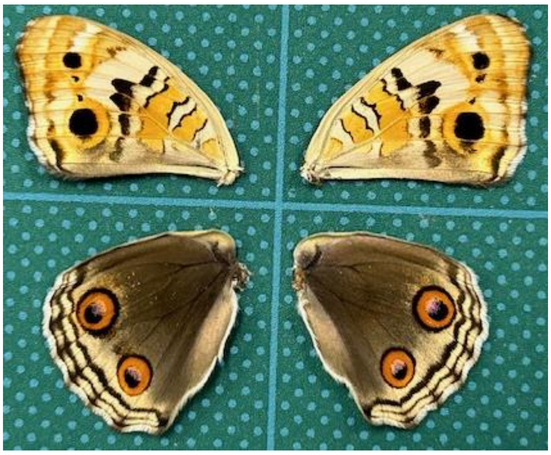

NO30

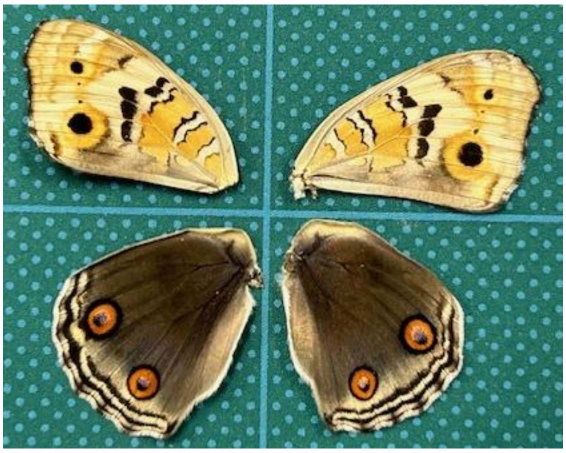

NO31

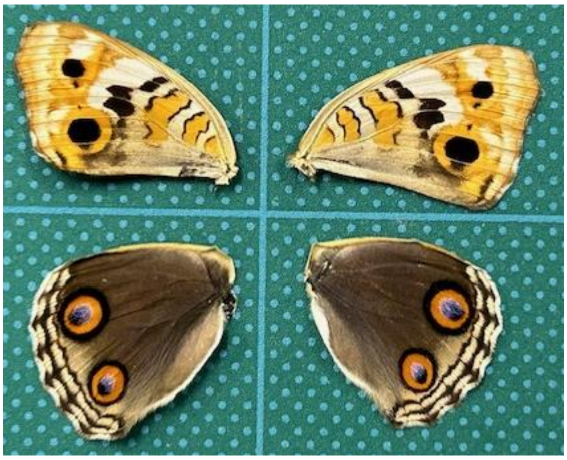

NO32

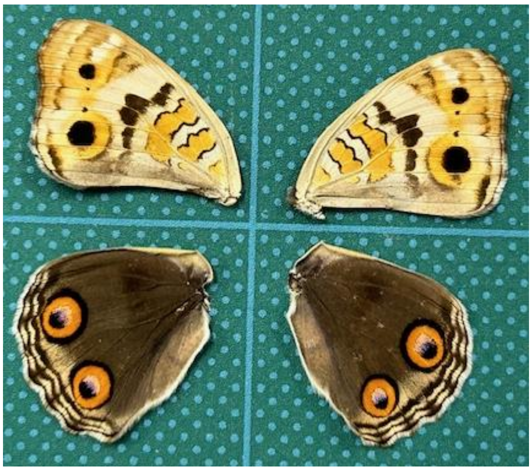

NO33

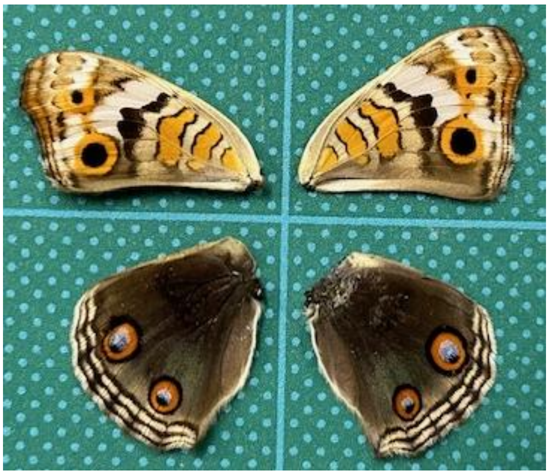

NO34

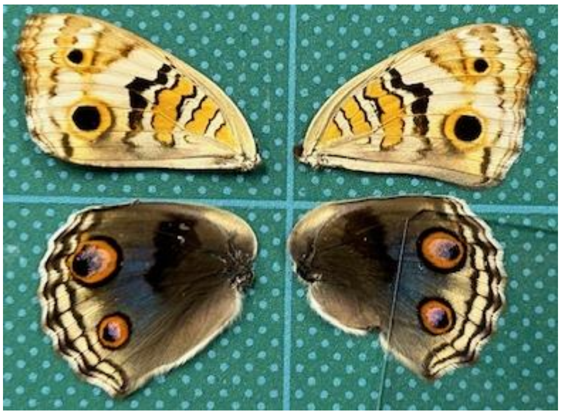

NO35

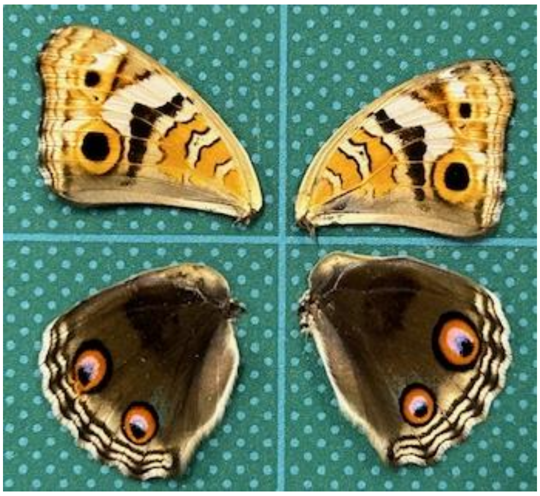

NO36

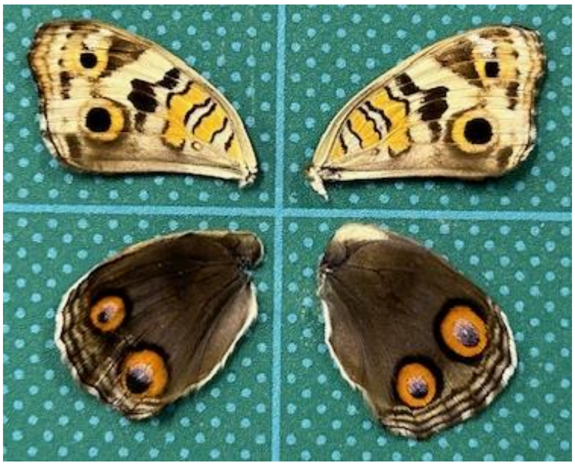

NO37

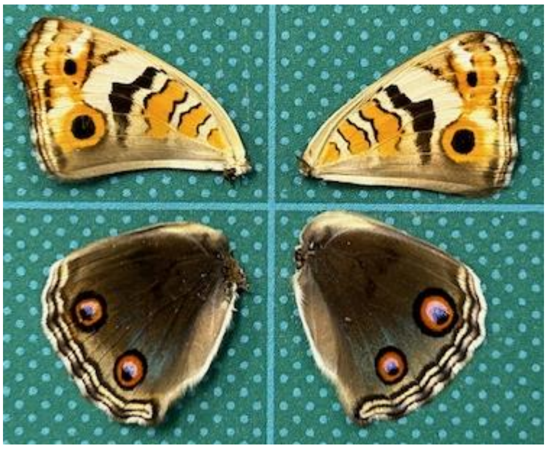

NO38

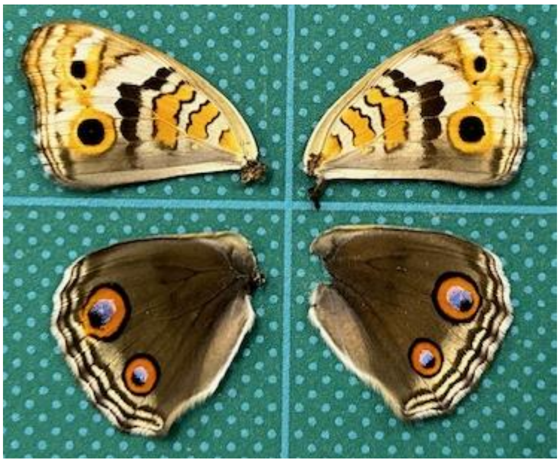

NO39

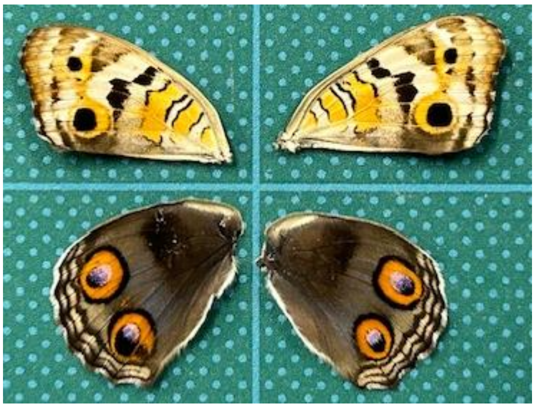

NO40

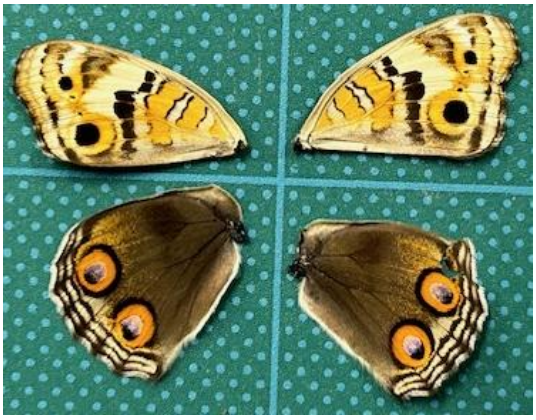

NO41

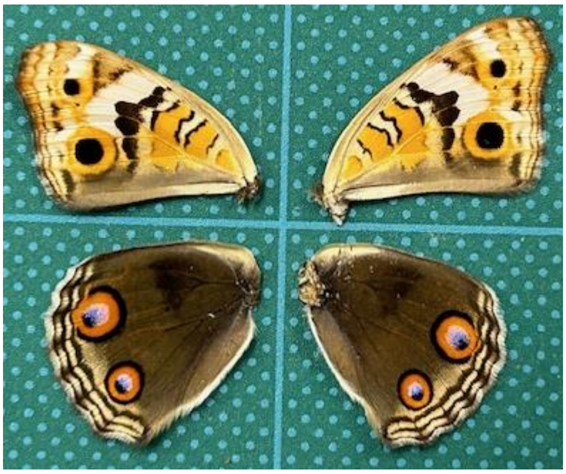

NO42

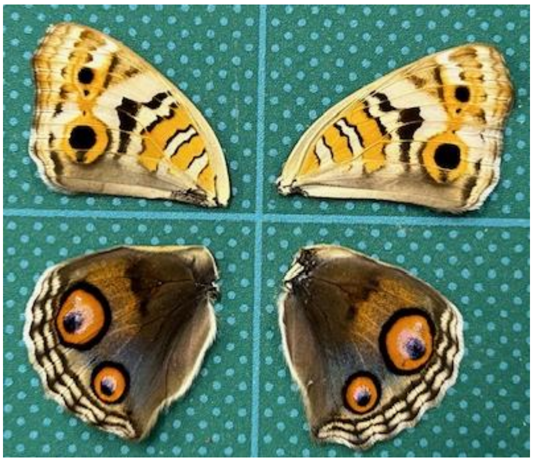

NO43

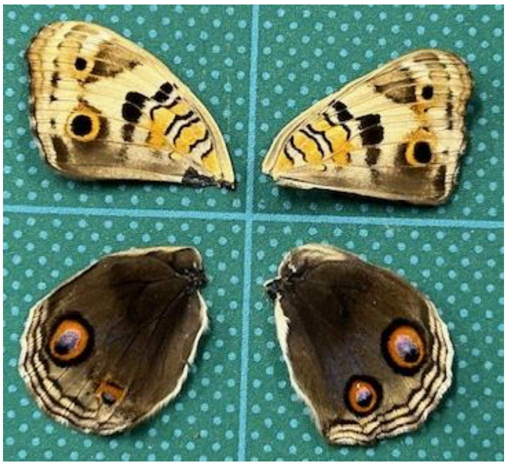

NO44

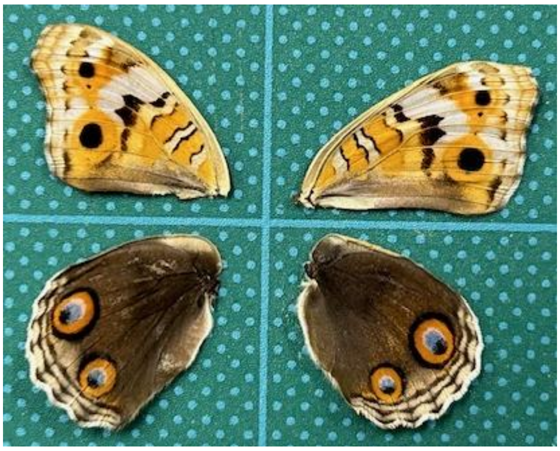

Summary: Anti-Dll antibody, Sandwich, Female

<u>TOTAL number of successful eclosion = 44</u>

PFE pulling (invagination) in an eyespot-less compartment:

No.21, No.23, No.24, No.25 (*n* = 4)

Ectopic black and orange band/spot between eyespots:

No.2, No.12, No.16, No.26, No.29 (black only), No.36 (black only) (*n* = 6)

Loss of PFE (including partial loss):

No.17, No.27, No.28, No.30, No.35 (redundant) (*n* = 5)

PFE pulling (invagination) in an eyespot-existing compartment:

No.4, No.5, No.35 (redundant) (*n* = 3)

<u>TOTAL number of modified individuals (excluding size change) = 17</u>

Note: In these images, the distance between the centers of adjacent dots is 1.5 mm.
